# Heterologous expression of the insect SVWC peptide WHIS1 inhibits *Candida albicans* invasion into A549 and HeLa epithelial cells

**DOI:** 10.3389/fmicb.2024.1358752

**Published:** 2024-05-30

**Authors:** Ming Chen, Wei-Kang Huang, Yang Yao, Shi-Mei Wu, Yong-Xin Yang, Wen-Xia Liu, Gang Luo, Shao-Feng Wei, Hua Zhang, Hong-Mei Liu, Bing Wang

**Affiliations:** ^1^Engineering Research Center of Health Medicine Biotechnology of Guizhou Province & School of Biology and Engineering (Modern Industry College of Health Medicine) & School of Public Health, Guizhou Medical University, Guiyang, Guizhou, China; ^2^Key Laboratory of Environmental Pollution Monitoring and Disease Control, China Ministry of Education (Guizhou Medical University), Guiyang, Guizhou, China; ^3^School of Basic Medical Science, Guizhou Medical University, Guiyang, Guizhou, China; ^4^Department of Laboratory Medicine, Guizhou Provincial People's Hospital, Affiliated Hospital of Guizhou University, Guiyang, Guizhou, China

**Keywords:** AMP, *C. albicans*, antimicrobial activity, WHIS1, hypha

## Abstract

*Candida albicans* (*C. albicans*), a microbe commonly isolated from *Candida vaginitis* patients with vaginal tract infections, transforms from yeast to hyphae and produces many toxins, adhesins, and invasins, as well as *C. albicans* biofilms resistant to antifungal antibiotic treatment. Effective agents against this pathogen are urgently needed. Antimicrobial peptides (AMPs) have been used to cure inflammation and infectious diseases. In this study, we isolated whole housefly larvae insect SVWC peptide 1 (WHIS1), a novel insect single von Willebrand factor C-domain protein (SVWC) peptide from whole housefly larvae. The expression pattern of WHIS1 showed a response to the stimulation of *C. albicans*. In contrast to other SVWC members, which function as antiviral peptides, interferon (IFN) analogs or pathogen recognition receptors (PRRs), which are the prokaryotically expressed MdWHIS1 protein, inhibit the growth of *C. albicans*. Eukaryotic heterologous expression of WHIS1 inhibited *C. albicans* invasion into A549 and HeLa cells. The heterologous expression of WHIS1 clearly inhibited hyphal formation both extracellularly and intracellularly. Furthermore, the mechanism of WHIS1 has demonstrated that it downregulates all key hyphal formation factors (*ALS1, ALS3, ALS5, ECE1, HWP1, HGC1, EFG1*, and *ZAP1*) both extracellularly and intracellularly. These data showed that heterologously expressed WHIS1 inhibits *C. albicans* invasion into epithelial cells by affecting hyphal formation and adhesion factor-related gene expression. These findings provide new potential drug candidates for treating *C. albicans* infection.

## Introduction

Candidal vaginitis is a commonly observed gynecological disease in the clinic (Agrawal et al., 2023), and vulvovaginal candidiasis (VVC) affects women worldwide (Hong et al., 2022; Kan et al., 2022; Schwebke et al., 2022). The microbe *Candida albicans* (*C. albicans*) is commonly isolated from patients with vaginal tract infections (Roudbarmohammadi et al., 2016; Esfahani et al., 2022; Wagner et al., 2022) and is also normally present in the oral cavity and intestines (Mayer et al., 2013; Gao et al., 2019). Thus, this pathogen increases the occurrence of opportunistic mucosal infections, especially in immunocompromised individuals. According to many reports, *C. albicans* is becoming increasingly resistant to many antifungal agents, such as ketoconazole and amphotericin (Hong et al., 2022; Nyirjesy et al., 2022; Agrawal et al., 2023), possibly due to the potential formation of mycelia and biofilms that protect it against antibiotic treatment (Moyes et al., 2015; Gulati and Nobile, 2016; Kadosh, 2016).

*C. albicans* hyphae are key pathogenetic factors that differ between yeast cells and mycelia and are regulated by several networks (Fan et al., 2013; Jenull et al., 2017; Desai et al., 2018). In yeast form, cells can flow with the host digestive tract and migrate, exhibiting decreased pathogenicity in patients (Cleary et al., 2011; Mayer et al., 2013; Moyes et al., 2015). In hyphal form, *C. albicans* produces many mycelia and wraps together (Mayer et al., 2013; Moyes et al., 2015; Jenull et al., 2017). Mycelia readily adhere to host infection sites and form biofilms for long-term persistence in the host (da Silva Dantas et al., 2016; Gulati and Nobile, 2016). The genes encoding agglutinin-like sequences (*ALS1, ALS3*, and *ALS5*) promote the adhesion of *C. albicans* (Fan et al., 2013; Roudbarmohammadi et al., 2016; Chevalier et al., 2019). *ALS3*, a cell surface glycoprotein, promotes adhesion and biofilm formation and affects *ALS1* and *ALS5* (Cleary et al., 2011; Fan et al., 2013; Roudbarmohammadi et al., 2016). Secreted aspartyl proteinase 6 (*SAP6*) participates in maintaining the cell surface integrity of *C. albicans* (Buu and Chen, 2013). Moreover, the extent of cell elongation 1 (*ECE1*) and hyphal wall protein 1 (*HWP1*), which are hypha-specific genes and hyphal morphological switches, depend on the hyphal formation (Nobile et al., 2006; Fan et al., 2013; Richardson et al., 2018; Liu et al., 2021). In addition, hyphal G cyclin 1 (*HGC1*) and enhanced filamentous growth transcription factor (*EFG1*), which are major activators of hyphal development, regulate hypha-inducing signals (Fan et al., 2013; Panariello et al., 2017; Desai et al., 2018). Zinc-responsive activator protein (*ZAP1*) governs the balance between hyphal cells and yeast cells in the biofilms of *C. albicans* (Nobile et al., 2009; Ganguly et al., 2011).

Houseflies living in complex environments rely on their powerful innate immune system (Wang et al., 2020). Antimicrobial peptides (AMPs) play an important role in protecting insects against environmental pathogens via the regulation of the toll signaling pathway (Gao et al., 2015; Wang et al., 2020). Hence, insects represent a rich source for the discovery of new AMPs. Many studies have confirmed the biological functions of AMPs, such as their anticancer (Bao et al., 2021; Timmons and Hewage, 2022), antiviral (Liu R. et al., 2022; Nabeta et al., 2022; Nikyar et al., 2022), antibacterial (Maleki Dizaj et al., 2022; Wang et al., 2022b) and immunoregulatory activities (Liu et al., 2018; Liu H. et al., 2022). There are also some reports on the anti-*C. albicans* activity of AMPs (Hu et al., 2022; Nabeta et al., 2022; Peng-Wei et al., 2022).

The single von Willebrand factor C-domain protein (SVWC) family contains many members that are always found in arthropods (Wang et al., 2017). *Macrobrachium nipponense* SVWC reportedly functions as an extracellular pattern recognition receptor (PRR) in river prawns (Qin et al., 2020) and helps the host defend against white spot syndrome virus (WSSV) (Nan et al., 2023). *Eriocheir sinensis* SVWC is involved in antibacterial responses in the Chinese mitten crab as a PRR (Qin et al., 2022). Simultaneously, the SVWC peptide of *Marsupenaeus japonicus* Vago functions as an IFN analog with anti-WSSV activity via the JAK-STAT signaling pathway (Gao et al., 2021). Moreover, SVWC members also exist in insects. *Drosophila melanogaster* Vago (*Dm*Vago), as an insect SVWC, is reportedly an antiviral peptide (Gao et al., 2021), and *Culex quinquefasciatus* Vago (*Cq*Vago) functions as an IFN analog with anti-WNV activity (West Nile virus) (Paradkar et al., 2012). Most importantly, Zhu et al. identified 29 SVWC peptides in houseflies as AMPs using bioinformatic methods (Qi et al., 2021). Thus, the housefly SVWC peptide may have great potential as an AMP.

In this study, we identified an insect SVWC peptide, viz. WHIS1, from houseflies that inhibits the growth of *C. albicans*. Both heterologous prokaryotic and eukaryotic expressions of the WHIS1 protein could limit the growth of *C. albicans*. Furthermore, WHIS1 affected *C. albicans* invasion into A549 and HeLa cells. In addition, heterologous eukaryotic expression of WHIS1 inhibited hyphal formation and adhesion-related gene expression in A549 and HeLa cells both extracellularly and intracellularly. These findings also provide new candidates and a theoretical basis for the clinical treatment of *C. albicans* infection.

## Materials and methods

### Microbial strains and culture conditions

The bacterial strains (*Staphylococcus aureus* ATCC 25923*, Escherichia coli* ATCC 25922) were cultured overnight in tryptic soy broth (TSB) or Mueller Hinton broth (MHB) at 37°C and 200 rpm. *C. albicans ATCC10231* was precultured overnight at 37°C with shaking at 200 rpm in potato dextrose broth (PDB) and cultured in potato-dextrose agar (PDA) at 28 or 37°C according to the different experimental requirements.

### Cell culture

Human lung carcinoma A549 cells were kindly provided by the School of Public Health, Guizhou Medical University. Human cervical cancer cells (HeLa cells) were kindly gifted by the School of Biology and Engineering (Modern Industry College of Health Medicine). The A549 cells and HeLa cells were maintained and grown to confluence in 25 cm^2^ cell culture flasks containing Dulbecco's modified Eagle's medium (DMEM, Gibco) supplemented with 10% fetal bovine serum (FBS, Gibco) and 100 μg/ml penicillin–streptomycin at 37°C in an atmosphere with 5% CO_2_. Once the cells had grown to 80% confluence in the culture flasks, they were detached with a solution containing 0.25% trypsin-0.53 mM EDTA (Gibco) and then seeded into six-well culture plates (5 × 10^5^ cells/well) or 24-well culture plates (8 × 10^4^ cells/well) in preparation for transfection and subsequent experiments without antibiotics (in DMEM).

### cDNA cloning and sequence analysis and structural modeling of WHIS1

The WHIS1 gene and its homologous version WHISIM were amplified using PCR from the *C. albicans*-stimulated housefly 3rd larval cDNA template, and the sequences were subsequently submitted to NCBI GenBank under project SUB11062418 (GenBank accession number SUB11062418 *Musca domestica WHIS1*: OQ267400; *M. domestica WHIS1* mutant: OQ267401). The open reading frames (ORFs) were analyzed by ORF Finder in DNAMAN. The ORFs without nucleotide sequences coding for a signal peptide of WHIS1 and its mutant were then cloned and inserted into the pET29a (+) vector for prokaryotic expression. The full-length ORF of WHIS1 was cloned and inserted into the PcDNA3.1(+) vector with the KpnI and NotI cleavage sites for eukaryotic expression. The signal peptide was analyzed using SignalP 5.0 online software (SignalP - 5.0 - Services - DTU Health Tech). Whole-ORF amino acid sequences were utilized for protein BLAST analysis. We set a target maximum of 1,000 sequences but a homologous sequence target of only 119 (Supplementary Excel 1 contains the BLAST details and accession number). The phylogenetic tree was constructed with NCBI Tree Viewer online software. The structural model of WHIS1 was generated with Phyre 2 online software. The amino acid sequence of WHIS1 was analyzed using the Helical Wheel Projection Program (http://rzlab.ucr.edu/scripts/wheel/wheel). The pI was predicted with ProtParam online software. Amino acid sequence alignment of MdWHIS1 with its homologs was performed using BioEdit software. The phylogenetic tree was subsequently constructed using MEGA software with the N-J method, and 1,000 bootstraps were generated.

### Prokaryotic expression and protein purification of WHIS1

The WHIS1 ORF without the nucleotide sequence encoding the N-terminal 20-amino-acid signal peptide was cloned and inserted into the pET29a (+) prokaryotic expression vector between the NdeI and XhoI restriction endonuclease sites. The host cells, *E. coli* BL21(DE3), were cultured in LB liquid medium at 37°C and 200 rpm overnight. First, the liquid cultures were transferred into a new culture (1:1,000 volume) and incubated for 2–3 h until the OD600 value reached 0.4–0.6. Subsequently, the 0.2 mM IPTG was added, and the bacteria were cultured at 16 or 25°C for 24 h. IPTG was used to induce the prokaryotic expression of WHIS1. Then, the bacteria were collected from cultured liquid medium by centrifugation at 12,000 rpm. After washing with PBS 3 times, the bacteria cells were disrupted by hypothermic ultrasonication. Then, the lysed cells were centrifuged at 12,000 rpm at 4°C. Then, the cell lysate could be divided into supernatant and precipitate. These samples were analyzed by SDS–PAGE. The proteins in the supernatant samples were soluble proteins, and the proteins in precipitates consisted of inclusion bodies, which are usually not easily soluble in PBS or water. After a large number of WHIS1 inclusion bodies was obtained, 8M urea was utilized to make it soluble in 50 mM Tris buffer. Then, urea dialysis was performed with a dialysis bag. The urea concentration was reduced from 8 to 6, 5, 4, and 2 M, among other concentrations, in a urea buffer for gradient reduction. The His•Bind^®^ Purification Kit (EMD Millipore Corp., Billerica, MA, USA) was subsequently used for protein purification according to the manufacturer's protocol. Subsequently, 30- and 15-kDa ultrafiltration devices (tube + membrane chips) from Millipore were used for further protein filtration. After centrifuging at 10,000 rpm at 4°C, the molecular weight of proteins less than the membrane pore size will be filtered into the bottom tube, and the molecular weight of the protein bigger than the membrane pore size will remain on the membrane chip of the ultrafiltration tube. The eluted protein mixture solution was first filtered through a 30-kDa ultrafiltration tube, and the filtrate was subsequently filtered through a 15-kDa ultrafiltration tube. The resulting filtrate was used for the minimum inhibitory concentration (MIC) test, and a time–growth curve was constructed. The homogeneity of the final preparations was analyzed by SDS–PAGE.

### Western blot assay

As described above, WHIS1 without its signal peptide was fused with His Taq in pET29a(+). After inducing with IPTG for prokaryotic expression and purification of WHIS1 protein, different samples were treated by boiling with RIPA buffer. Then 25 μl of the sample was loaded into a gel well. After completing SDS–PAGE, the gel was transformed into a PVDF membrane. Then, the membrane was sealed with milk for 2 h. Then, the membranes were incubated with anti-His antibody at 4°C for 12 h. After incubation, the membranes were washed with TBST buffer for three times, for 10 min each time. The second antibody was incubated for 2 h. After washing with TBST another three times, ECL chemical kits were utilized for the development of chemiluminescent color in the BIORAD Scanner. Thus, we also detected the protein by Western blotting with the anti-His antibody of WHIS1.

### Antimicrobial activity analysis and time–growth curve

The minimum inhibitory concentration (MIC) and minimum fungicidal concentration (MFC) were determined by the microbroth dilution method (Wang et al., 2021a,b) according to the conditions recommended by the Clinical and Laboratory Standards Institute (CLSI, a reference method for broth dilution antifungal susceptibility testing of yeasts, in Approved Standard M27-A3, third ed., Clinical and Laboratory Standards Institute, Wayne, PA, USA, 2008). A single colony of *C. albicans* was placed in a PDB medium and was cultured overnight (*E. coli* and *S. aureus* in TSB or MHB). WHIS1 and its mutant were obtained by prokaryotic expression at a concentration of 2–250 μg/ml in a sterile RPMI 1640 medium (Gibco). Then, 100 μl (5 × 10^7^ CFU/ml) of working *C. albicans* solution was added to each well, and the final total volume in each well was 200 μl. The samples were incubated on a shaker at 150 rpm and 37°C for 12 h. The MIC and MFC were visually determined, and PDA plate diffusion verification was performed (Wang et al., 2021a,b). The kinetics of the anti-*C. albicans* activity of prokaryotically expressed WHIS1 and its mutant were studied. A 96-well plate containing a suspension (a final concentration of 5 × 10^7^ CFU/ml) and PDB containing different concentrations of prokaryotically expressed WHIS1 and its mutant was incubated at 28°C, and equal amounts of medium were used as the solvent control. A microplate reader was subsequently used to measure the OD_595nm_ value of the sample at different time points, and the OD_595nm_ value was used as the ordinate to construct the time–growth curve.

### Cell transfection procedures

Lipofectamine 2000 reagent was used for transfection according to the manufacturer's instructions. A549 and HeLa cells were divided into two groups: the *WHIS1* group and the vector group. The culture medium was replaced with Opti-MEM before transfection. The samples for the six-well plates were prepared as follows: the plasmid (4 μg) was mixed in 200 μl of Opti-MEM and incubated for 5 min, and in a separate tube, the liposomes (10 μl) were suspended in 200 μl of Opti-MEM and incubated for 5 min. The samples for the 24-well plates were prepared as follows: the plasmid (1 μg) was mixed in 50 μl of Opti-MEM and incubated for 5 min, and in a separate tube, the liposomes (2.5 μl) were suspended in 50 μl of Opti-MEM and incubated for 5 min. Both the plasmid and liposomes in Opti-MEM were mixed gently by pipetting and incubated for 15 min at room temperature. The cells were seeded in 6- or 24-well culture plates, allowed to adhere for 24 h, and transfected with DNA-liposome complexes. The various mixtures were added carefully to each well of the cell culture plates, and the plates were then shaken gently. The plates were incubated at 37°C and 5% CO_2_ for 5 h, the medium was replaced with DMEM containing 10% FBS, and various experiments were subsequently conducted.

### Toxic assay of amphotericin b to A549 cells

A549 cells were seeded with a density of 5,000 cells/well in 96-well plates containing the DMEM medium (10% FBS) overnight at 37°C in an atmosphere with 5% CO_2_. The cells were treated with different concentrations (30μg/ml, 35μg/ml, 40μg/ml, and 45μg/ml) of amphotericin B diluted in DMEM medium and were incubated for different times (20 min, 40 min, 1 h, and 24 h). Subsequently, 100 μl of 10% CCK8 solution was added to the cells and incubated for 1 h. Then the absorbance was read at a 450 nm test wavelength using a microplate reader.

### Hyphal formation assay of *C. albicans*

Twenty-four hours after the transfection of A549 or HeLa cells with WHIS1 or vector, *C. albicans* suspensions were calibrated in RPMI 1640 with 10% FBS, and the transfected cells were infected at a multiplicity of infection (MOI) of 20 for 8 h at 37°C and 5% CO_2_. The *C. albicans* suspensions were used to calculate cell number (1.6 × 10^6^ CFU/ml) according to the McFarland method (0.5). The MOI was calculated by the *C. albicans* cell number divided by the host cell number (A549/HeLa). After incubation, the vector and WHIS1 groups were observed using Cytation 5 at different magnifications under the same view field. This procedure was used for the total observation of all *C. albicans* specimens. After the supernatant was discarded and the cells were washed twice with PBS, Cytation 5 was used to capture images of *C. albicans* attached to the cell surface at different magnifications. Subsequently, RPMI 1640 containing 40 μg/ml of amphotericin B was added to kill the *C. albicans* cells outside the monolayer. The plate was incubated at 37°C in 5% CO_2_ for 20 min and washed twice with PBS. The monolayer cells were digested with 0.25% trypsin-0.53mM EDTA and subsequently lysed with 0.1% Triton X-100 for 10 min in a cell incubator. The two groups were subsequently photographed with Cytation 5 at different magnifications (Peng-Wei et al., 2022). This process was used for the analysis of *C. albicans* invasion into the cells.

### Effect of WHIS1 on *C. albicans* invasion into A549 and HeLa cells: different MOIs

Before their use in the invasion assays, the cells were washed with phosphate-buffered saline (PBS). *C. albicans* suspensions were calibrated in DMEM supplemented with 10% FBS, and the transfected cells were infected at an MOI of approximately 15 or 20 to infect for 8 h at 37°C and 5% CO_2_. Following incubation, each well was washed twice with 500 μl of PBS, and DMEM containing 40 μg/ml of amphotericin B was added to kill *C. albicans* outside the monolayer cells. The plate was then incubated at 37 °C in 5% CO_2_ for 20 min and washed twice with 500 μl of PBS. The monolayer cells were digested with 100 μl of 0.25% trypsin-0.53mM EDTA and subsequently lysed with 500 μl of 0.1% Triton X-100 for 10 min in a cell incubator. The *C. albicans* was subsequently plated on PDA plates to enumerate the intracellular fungi (CFU). Three wells were used for each tested group.

### Effect of WHIS1 on *C. albicans* invasion into A549 and HeLa cells: supernatant and intracellular microbes

Before their use in the invasion assays, the cells were washed with PBS. *C. albicans* suspensions were calibrated in DMEM supplemented with 10% FBS, and the transfected cells were incubated at an MOI of 20 for 8 h at 37°C and 5% CO_2_. After incubation, the supernatant of *C. albicans* was centrifuged at 3000 rpm for 3 min and resuspended in 600 μl of PBS. Serial dilution was then performed. Subsequently, 10 μl of each dilution was applied to a PDA plate and incubated at 37°C. For the collection of intracellular fungi, each well was washed twice with 500 μl of PBS, and DMEM containing 40 μg/ml of amphotericin B was added to kill *C. albicans* cells outside the monolayer. The plate was then incubated at 37 °C in 5% CO_2_ for 20 min and washed twice with 500 μl of PBS. The monolayer cells were digested with 100 μl of 0.25% trypsin-0.53 mM EDTA and subsequently lysed with 500 μl of 0.1% Triton X-100 for 10 min in a cell incubator. The *C. albicans* was then plated on PDA plates to enumerate the intracellular fungi (CFU). Three wells were used for each tested group.

### Expression analysis of the WHIS1 gene in houseflies or A549 and HeLa cells

Houseflies at different developmental stages, different organs of 3rd-instar housefly larvae or 3rd-instar larval samples, after exposure to different stimuli were ground with TRIzol reagent. RNA was then extracted according to the manufacturer's protocol. Similarly, total RNA from A549 and HeLa cells was extracted using TRIzol reagent at 1.5, 12, and 24 h after transfection and 8 h and 12 h after invasion. The RNA was reverse transcribed to cDNA using a PrimeScript™ RT Reagent Kit with a gDNA Eraser. Quantitative real-time PCR (RT–PCR) was used for the detection of gene expression with the qPCR master mix obtained from Biogoethe Biotechnology Co., Ltd, Wuhan. Housefly β*-actin* or GAPDH was selected as the internal reference gene for the assessment of the WHIS1 expression pattern by real-time PCR. Human β*-actin* and HeLa or A549 cells, 1.5 h after transfection, were used as an internal reference and control, respectively. The reaction process was carried out as follows: 95°C for 30 s followed by 40 cycles of 95°C for 10 s, 60°C for 30 s, and 72°C for 30 s. The 2^−ΔΔCt^ statistical method was used for data analysis (Wang et al., 2022a; Wang C. et al., 2022). The primers used are shown in Supplementary Table 2.

### Total RNA extraction and real-time PCR analysis of gene expression in *C. albicans*

*C. albicans* suspension was added to the transfected cells, and the MOI was adjusted to 20. After being allowed to invade A549 or HeLa cells for 8 h at 37°C in 5% CO_2_, the cells were centrifuged at 12,000 rpm for 5 min for sample collection. DEPC-treated water was used for the initial washes. For the collection of intracellular *C. albicans*, the supernatant was discarded, each well was washed twice with 1 ml of PBS, and DMEM containing 40 μg/ml of amphotericin B was added to kill *C. albicans* cells outside the monolayer. The plate was then incubated at 37°C in 5% CO_2_ for 20 min and washed twice with PBS. The monolayer cells were digested with 0.25% trypsin-0.53 mM EDTA and subsequently lysed with 0.1% Triton X-100 for 10 min in a cell incubator. Intracellular *C. albicans* was centrifuged at 12,000 rpm for 5 min for sample collection. Total RNA was extracted using the Fungal RNA kit and then reverse transcribed to cDNA using the PrimeScript™ RT Reagent Kit with a gDNA Eraser. Real-time PCR was utilized for the detection of gene expression. *18S* rRNA was selected as the internal reference gene. The reaction process and the statistical methods used were the same as those used for the abovementioned analyses. The sequences of the primers used are shown in Supplementary Table 3.

For the detection of target genes (WHIS1 and *C. albicans* genes) in different samples, including housefly larvae and organs and A549 and HeLa cells, different internal reference genes were selected. To detect the WHIS1 expression pattern in houseflies, GAPDH and β-actin were selected as the internal references for the tissue samples and the microbial stimulation samples (**Figure 4**), respectively, because these genes exhibit enhanced stability under the tested conditions, as described in previous reports (Gao et al., 2015). Human β-actin was selected as an internal reference for the detection of WHIS1 in A549 and HeLa cells (**Figures 6E**–**H**). *18S* rRNA was selected as an internal reference for *C. albicans* gene detection in A549 and HeLa cells to determine host cell gene expression (**Figures 8**, **9**). All the experimental procedures used in this study received ethical approval (no. 1702102) as described by Wang et al. (2021b). The statistical analyses were conducted using Microsoft Office Excel. Unpaired *t*-tests were used to compare two datasets. All the experiments were performed with three independent replicates.

## Results

### Characterization of WHIS1

**Figure 1:**
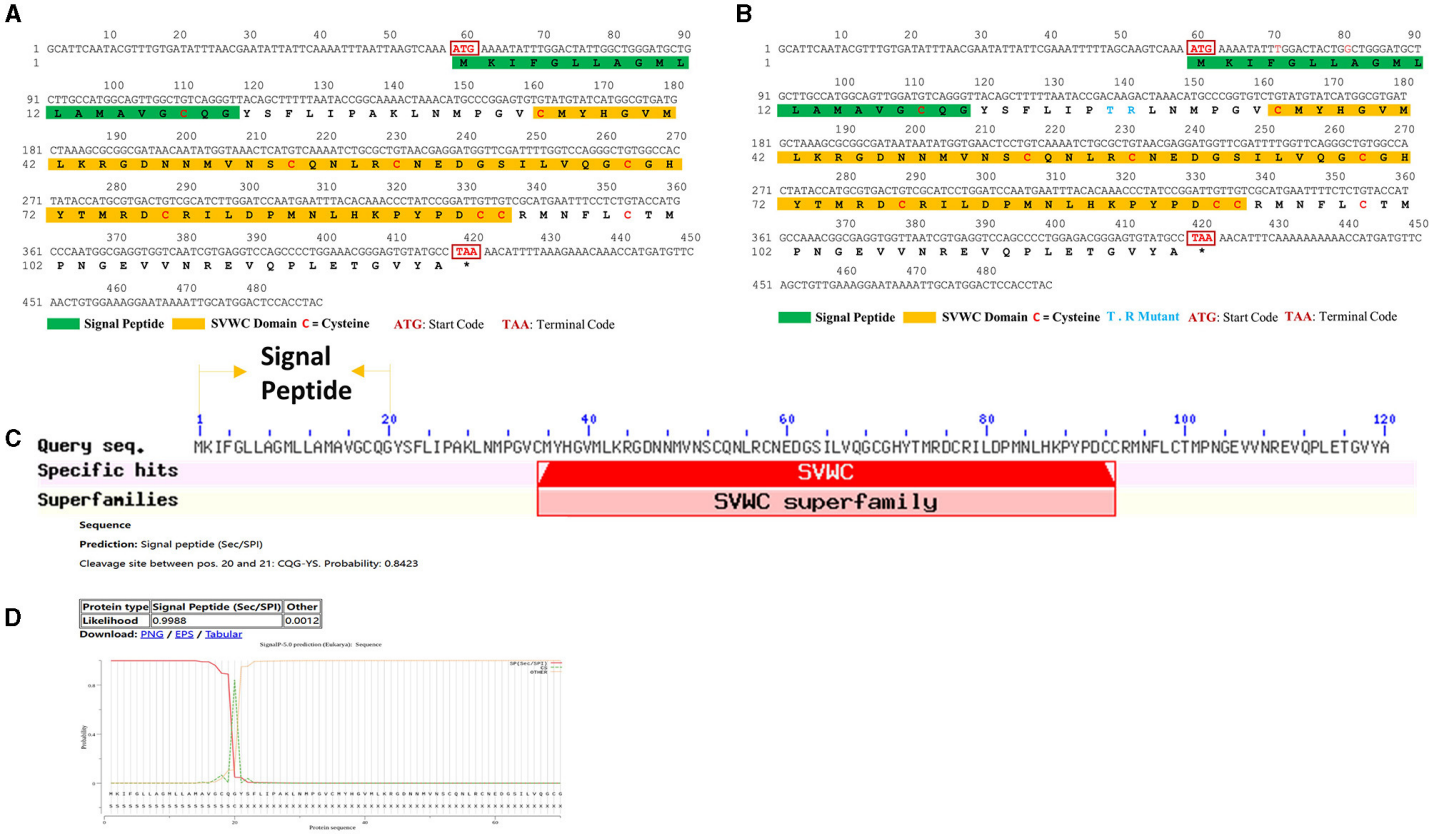
ORF and signal peptide of WHIS1. The ORFs of WHIS1 **(A)** or its mutant **(B)** were annotated with ORF Finder in DNAMAN. The signal peptide of WHIS1 was predicted by SignalP 5.0 **(D)**, and its domains were subjected to BLAST searches against the NCBI database **(C)**.

**Figure 2:**
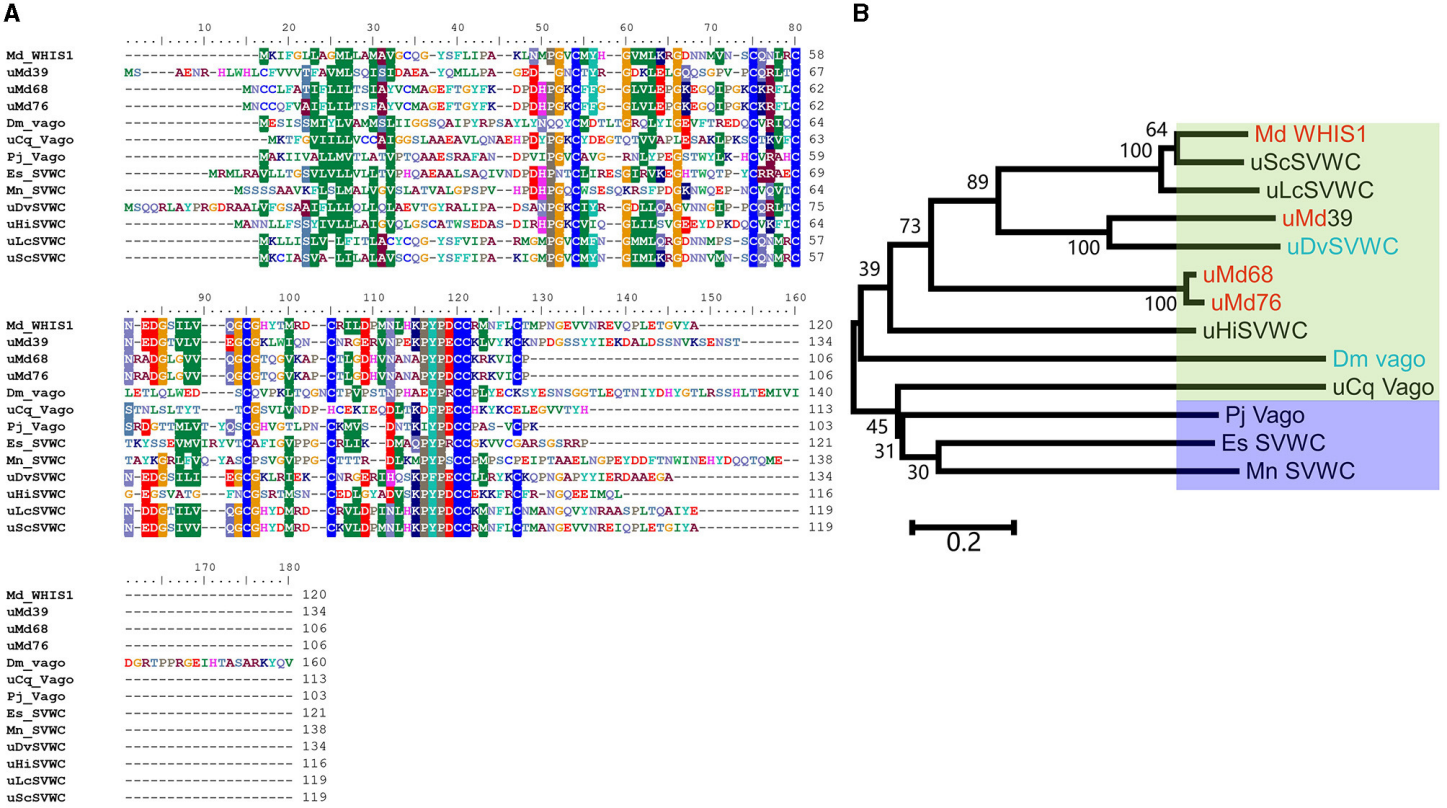
Sequence alignment and phylogenetic dendrogram of WHIS1 with other SVWC homologs. **(A)** Sequence alignment of *Musca domestica* WHIS1 with other SVWC homologs. **(B)** A phylogenetic tree was constructed using MEGA via the neighbor-joining tool with 100 bootstraps. The green background represents insects, the crystal represents arthropods, the red background represents *Musca domestica*, and the blue background represents *Drosophila*.

As shown in Supplementary Figure 1, WHIS1 was significantly upregulated in the microbe-stimulated housefly 3rd larval transcriptome. A BLAST analysis of the protein sequence against the NCBI database showed that this protein belongs to the single von Willebrand factor C-domain protein (SVWC) superfamily (Figures 1, 2). This protein has a signal peptide (the first 20 amino acids) and 8 conserved cysteines, including those in the SVWC domain (Figure 1A). The theoretical molecular weight of WHIS1 is 13,353.79. The pI of this protein is 7.52. Moreover, as revealed by SDS–PAGE, the protein was close to 12 kDa. Furthermore, proteins with high homology to the identified protein have been predicted but remain uncharacterized, as detailed in the NCBI database (Supplementary Figure 2 and Supplementary Excel 1). Thus, we named this peptide whole-housefly larvae insect SVWC peptide 1 (WHIS1). The antiviral peptide Vago in *D. melanogaster* and *Culex* mosquitoes, as an SVWC member, serves as an IFN analog to help the host combat viruses (Paradkar et al., 2012; Gao et al., 2021). In addition, other members of the SVWC family function as PRRs in arthropods such as *M. nipponense* and *E. sinensis* (Qin et al., 2020, 2022). Therefore, gene homology was analyzed by sequence alignment and the construction of a phylogenetic tree. Amino acid sequence alignment revealed that housefly WHIS1 was more similar to uncharacterized Md39 (*M. domestica*39), Md68, and Md76 compared to others (Figure 2A). Moreover, with the exception of 8 cysteines conserved in SVWC members, MdWHIS1 was not similar to *D. melanogaster* Vago (*Dm*Vago). A phylogenetic tree showed that housefly WHIS1 was more homologous to insect SVWCs compared to arthropod SVWCs. Furthermore, MdWHIS1 clustered with the SVWCs of *Stomoxys calcitrans* and *Lucilia cuprina* (Figure 2B). These data indicate that MdWHIS1 may have different functions in the fruit fly than those of Vago and arthropod SVWCs.

**Figure 3:**
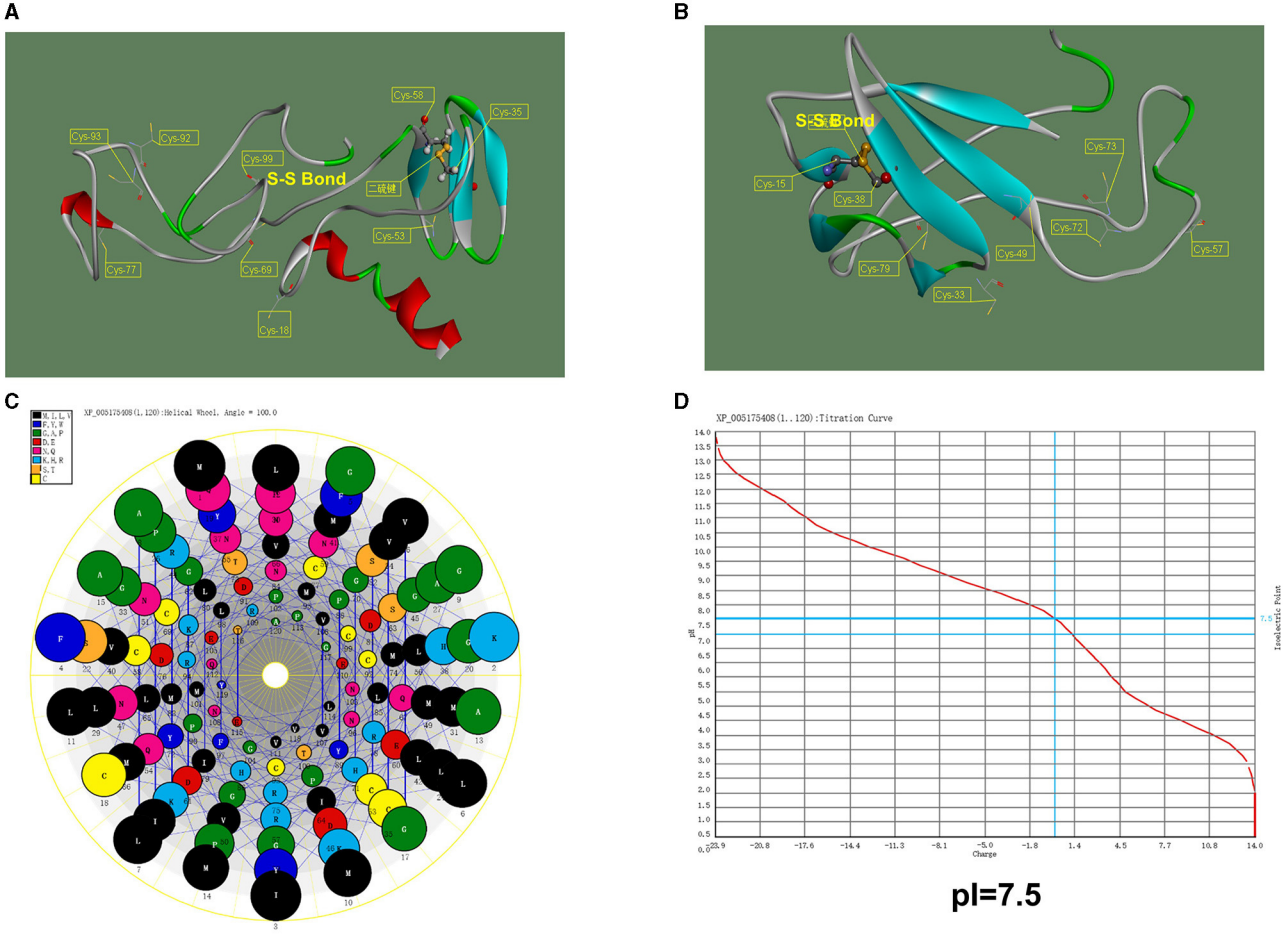
Structural model of WHIS1 and its mutant. Structural model of full-length WHIS1 with the signal peptide **(A)** and without the signal peptide **(B)**, prepared using Phyre 2 online software. **(C)** Helix wheel of the WHIS1 amino acids. **(D)** pI of WHIS1.

To further determine the function of the WHIS1, basic protein informatics analysis and structure mimicking were performed. As shown in Figure 3, the pI of WHIS1 is 7.52. Furthermore, structural modeling of WHIS1 showed that cysteine residues play an important role in the formation of internal S–S bonds to support the structure (Figures 3A, B). Moreover, we obtained a 2-amino-acid mutant or a variant of WHIS1 in houseflies (Figure 1B). Due to their high amino acid similarity, the proteins have similar properties.

### Expression pattern of WHIS1 in houseflies

**Figure 4:**
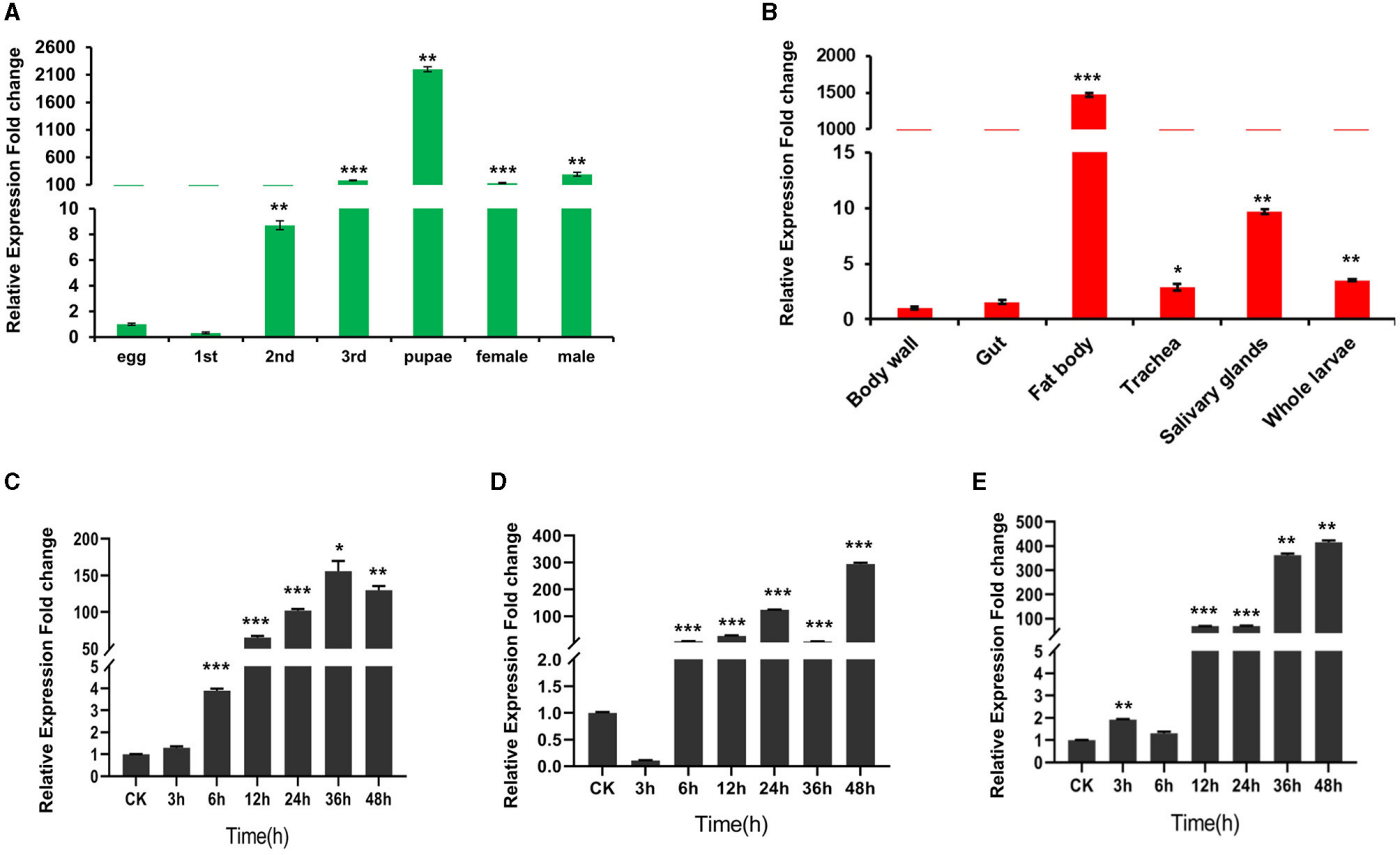
Expression pattern of WHIS1 in houseflies. **(A)** WHIS1 expression levels in houseflies at different developmental stages. **(B)** WHIS1 expression levels in different organs of 3rd-instar housefly larvae. WHIS1 expression levels in 3rd-instar housefly larvae after stimulation with *Escherichia coli* **(C)**, *Staphylococcus aureus* **(D)**, or *Candida albicans* **(E)**. *n* = 3, *P < 0.05, **P < 0.01, and ***P < 0.001, vs. the control group (first column).

As shown in Figure 4A, WHIS1 was highly expressed from the 2nd larval stage to the adult stage and was expressed at high levels mainly in the fat body and salivary glands (Figure 4B). In addition, the 3rd-instar larvae showed upregulated expression of WHSI after stimulation with *S. aureus, E. coli*, and *C. albicans* for 6 h or 12 h (Figures 4C–E). These findings indicate that WHIS1 may participate in the antimicrobial infection process in houseflies.

### Prokaryotic expression of WHIS1 and antimicrobial activity analysis

**Figure 5:**
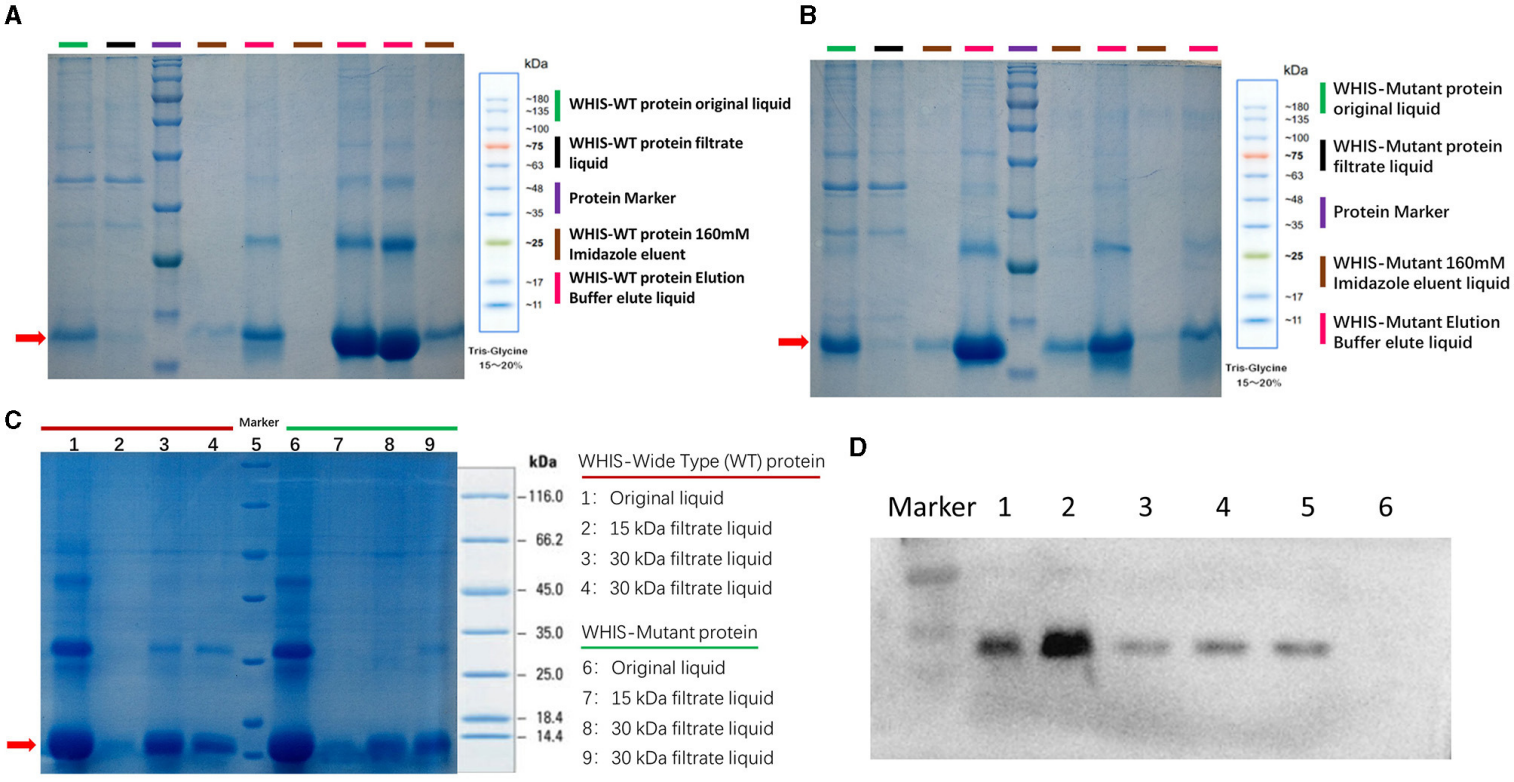
Prokaryotic expression of WHIS1 and its mutant. SDS–PAGE of purified wild-type **(A)** or mutant **(B)** WHIS1 protein or filtered protein **(C)**. **(D)** Western blotting of His antibody to prokaryotic WHIS1 with His Taq. Channel 1: 16°C induced prokaryotic WHIS1 supernatant, 2: WHIS1 precipitate, 3: Eluting WHIS1 with a buffer of 200 mM imidazole, 4: Eluting WHIS1 with 250 mM of imidazole buffer, 5: Purified WHIS1, 6: Un-induction sample.

To analyze the anti-*C. albicans* activity of WHIS1, the protein and its mutant was prokaryotically expressed. After purification of the protein, 30-kDa and 15-kDa ultrafiltration tubes were utilized to filter the elution mixture. The filtrate was used for the MIC test, and a time–growth curve was generated. All the samples were detected by SDS–PAGE (Figure 5). The MIC of WHIS1 was 0.011 mmol/L. The MIC and time–growth curves showed that WHIS1 and its mutant inhibited the growth of *C. albicans* (Supplementary Table 1 and Supplementary Figure 3).

### WHIS1 inhibits *C. albicans* invasion into A549 and HeLa cells

**Figure 6:**
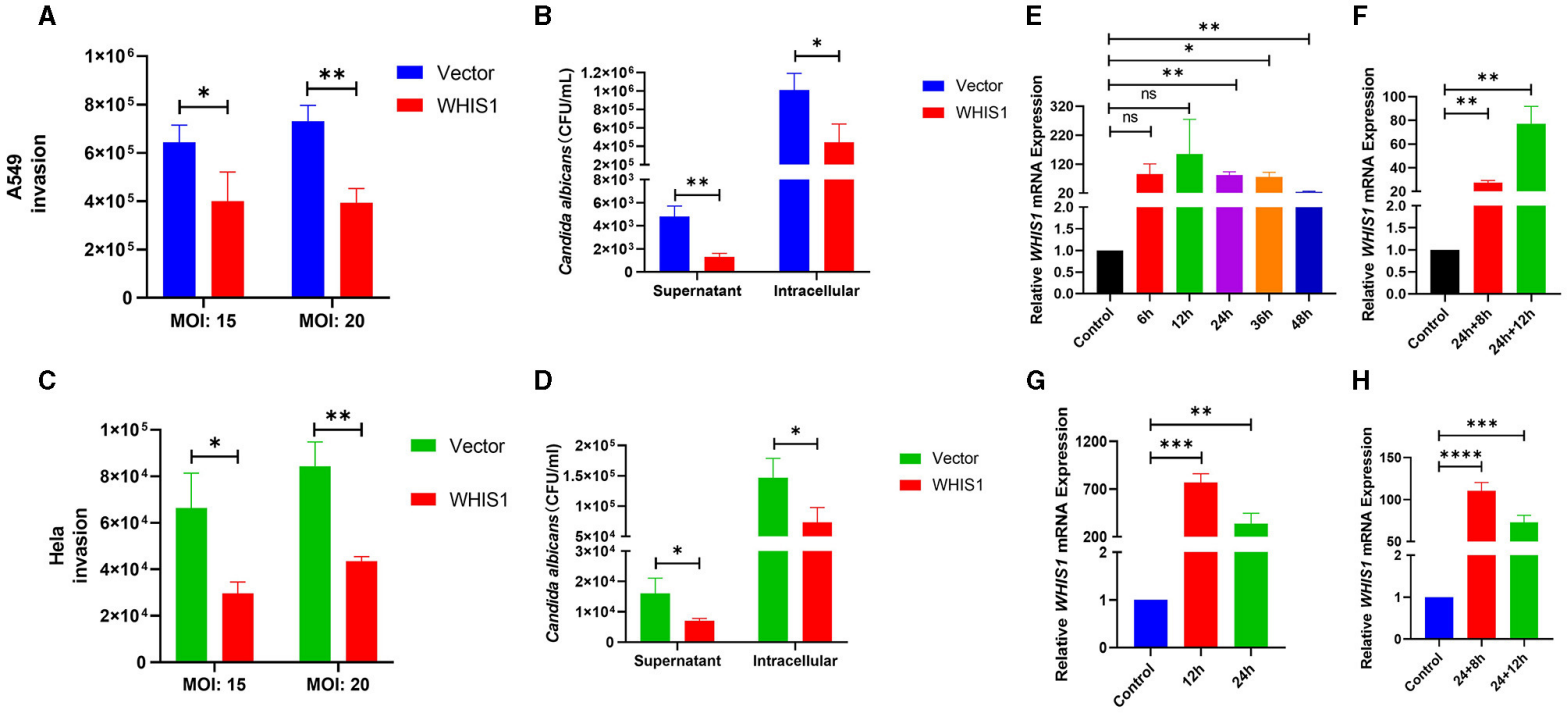
WHIS1 inhibits *Candida albicans* invasion into A549 and HeLa cells. Twenty-four hours after transfection with WHIS1, A549 **(A)** or HeLa **(C)** cells were infected with *C. albicans* at an MOI of 15 or 20, and the *C. albicans* CFUs were then calculated by the spread plate method. Similar to the abovementioned options, the supernatant or intracellular *C. albicans* CFUs in A549 **(B)** or HeLa **(D)** cells were counted. The WHIS1 expression level in A549 **(E)** or HeLa **(G)** cells at different time points post-transfection was measured. Using the same options as in **(B)**, the WHIS1 expression level in A549 **(F)** or HeLa **(H)** cells 24 h after transfection and infected with *C. albicans* for different time points was measured. *n* = 3, *P < 0.05, **P < 0.01, ***P < 0.001, and ****P < 0.0001 vs. the control group.

To further validate the anti-*C. albicans* activity of WHIS1, heterologous eukaryotic methods were used. WHIS1-PcDNA3.1(+) was transfected into A549 and HeLa cells for eukaryotic expression. Compared with the empty vector [PcDNA3.1(+)], WHIS1 decreased the invasion of *C. albicans* into A549 cells at an MOI of 15 or 20 (Figure 6A). Furthermore, both the supernatant and intracellular CFUs of *C. albicans* were reduced by transfection with WHIS1. Thus, WHIS1 can both inhibit cell invasion and limit the growth of *C. albicans* (Figure 6B). In addition, relatively high expression of WHIS1 was detected 24–36 h after transfection (Figure 6E). In addition, we detected the expression of WHIS1 after 8 h and 12 h of the same treatment similar to that used for the invasion experiment (Figure 6F). Both analyses showed that the WHIS1 expression level was high. Similar results were also observed in HeLa cells (Figures 6C, D, G, H). Overall, these data indicate that heterologous eukaryotic expression of WHIS1 inhibits *C. albicans* invasion and limits its growth.

### WHIS1 affects *C. albicans* hyphal formation

**Figure 7:**
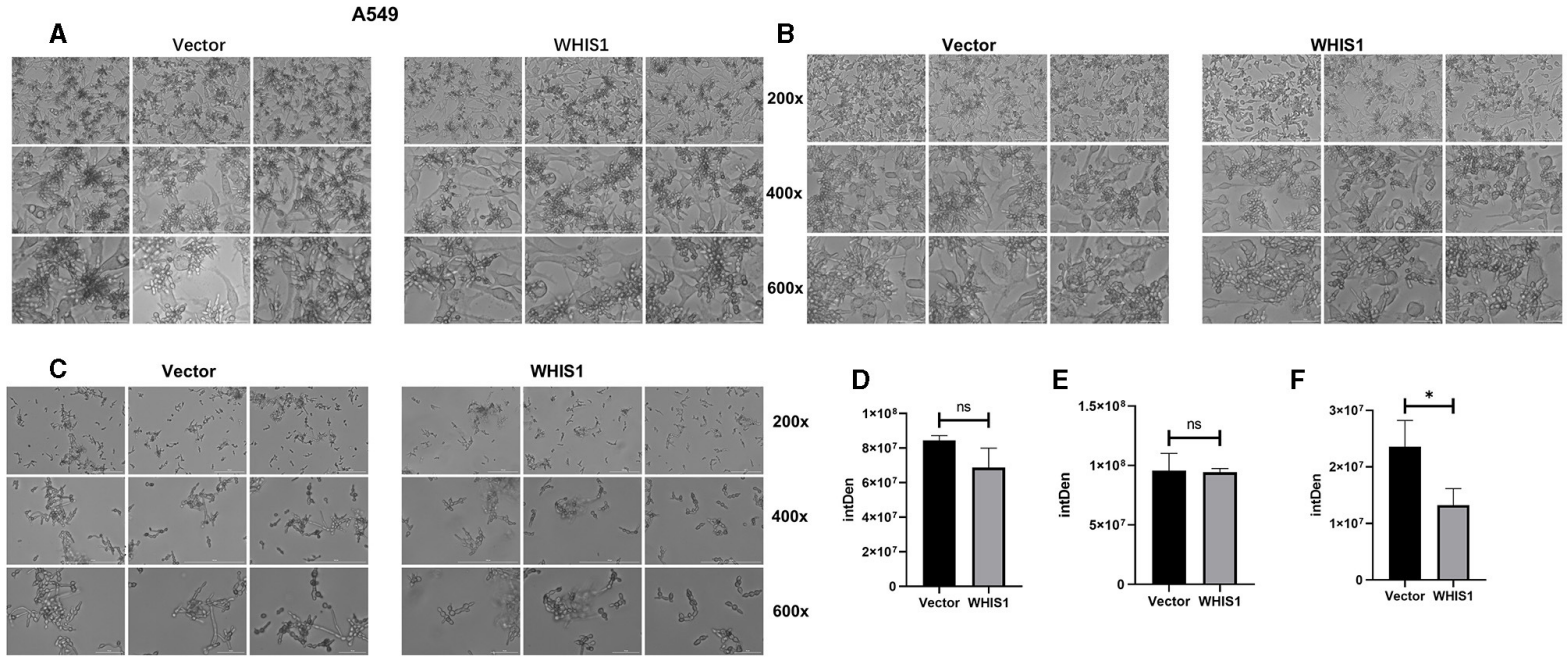
WHIS1 reduces the density and inhibits hyphal formation in *Candida albicans*-infected A549 cells. Morphological observation of *C. albicans* in medium **(A)**, attached to the surface of A549 cells **(B)**, after invading A549 cells **(C)**. IMAGE J software was used for gray density analysis, total **(D)**, on the surface **(E)**, and after invading cells **(F)**. Three replicate wells of each sample were observed with Cytation 5. *n* = 3, *P < 0.05, ns: not significant. vs. the control group.

**Figure 8:**
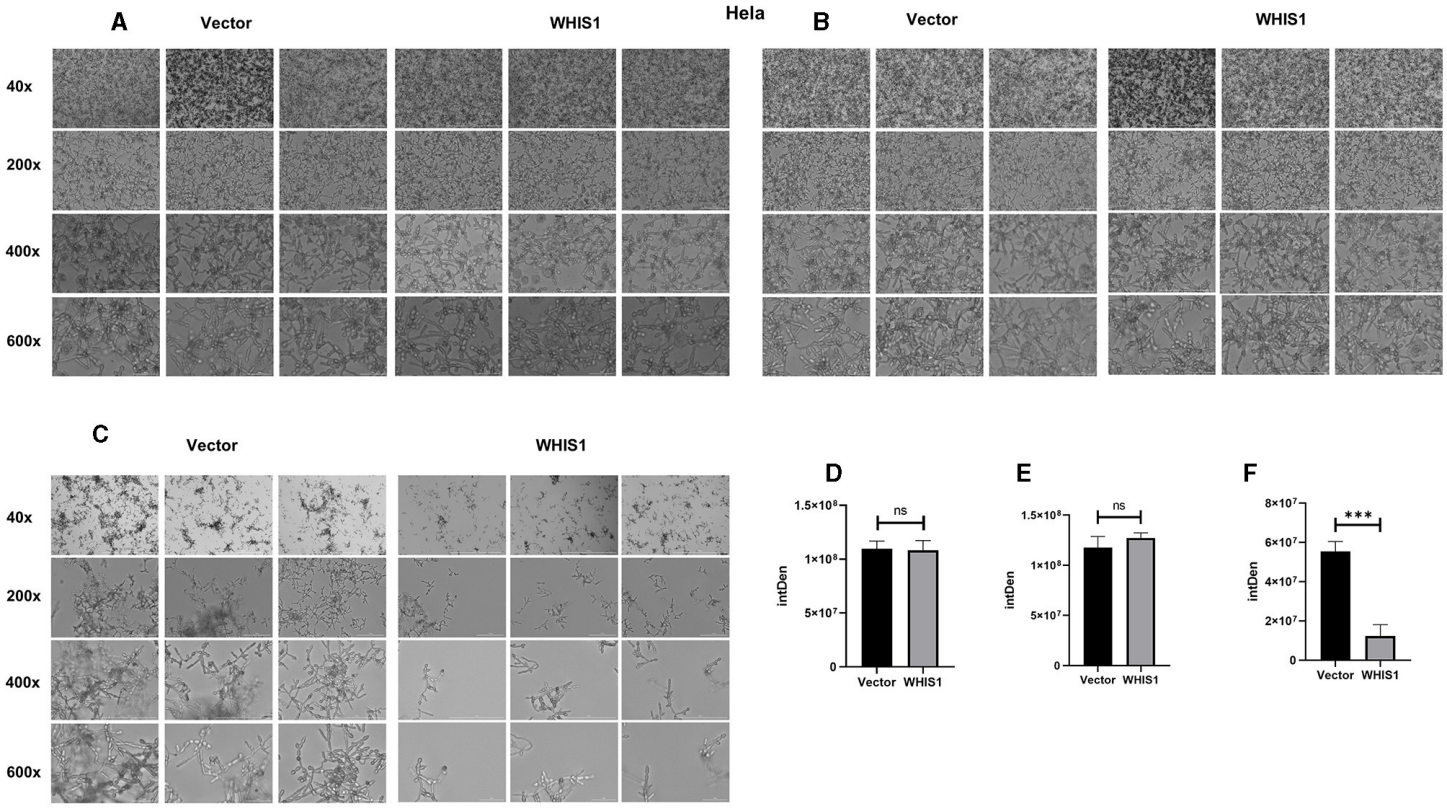
WHIS1 reduces the density and inhibits hyphal formation in *Candida albicans*-infected HeLa cells. Morphological observation of *C. albicans* in medium **(A)**, attached to the surface of HeLa cells **(B)**, after invading HeLa cells **(C)**. IMAGE J software was utilized for gray density analysis, total **(D)**, on the surface **(E)**, and after invading cells **(F)**. Three replicate wells of each sample were observed with Cytation 5. *n* = 3, ***P < 0.001, ns: not significant. vs. the control group.

Hyphal formation is a key factor in *C. albicans* invasion into host cells and its persistence at infection sites (Fan et al., 2013; Jenull et al., 2017; Nabeta et al., 2022). To further elucidate this phenomenon, a hyphal formation assay was performed using A549 and HeLa cells. The cell density and hyphal length of *C. albicans* were lower in the WHIS1 expression group compared with the vector group (Figures 7, 8). In addition, these phenomena were observed in the total supernatant (Figures 7A, D), on the cell surface (Figures 7B, E), and intracellularly (Figures 7C, F) in the A549. Similar data were observed in the total supernatant (Figures 8A, D), on the cell surface (Figures 8B, E), and intracellularly (Figures 8C, F) in the HeLa cells.

### WHIS1 affects *C. albicans* hyphae and biofilm-related gene expression and invasion into A549 and HeLa cells

**Figure 9:**
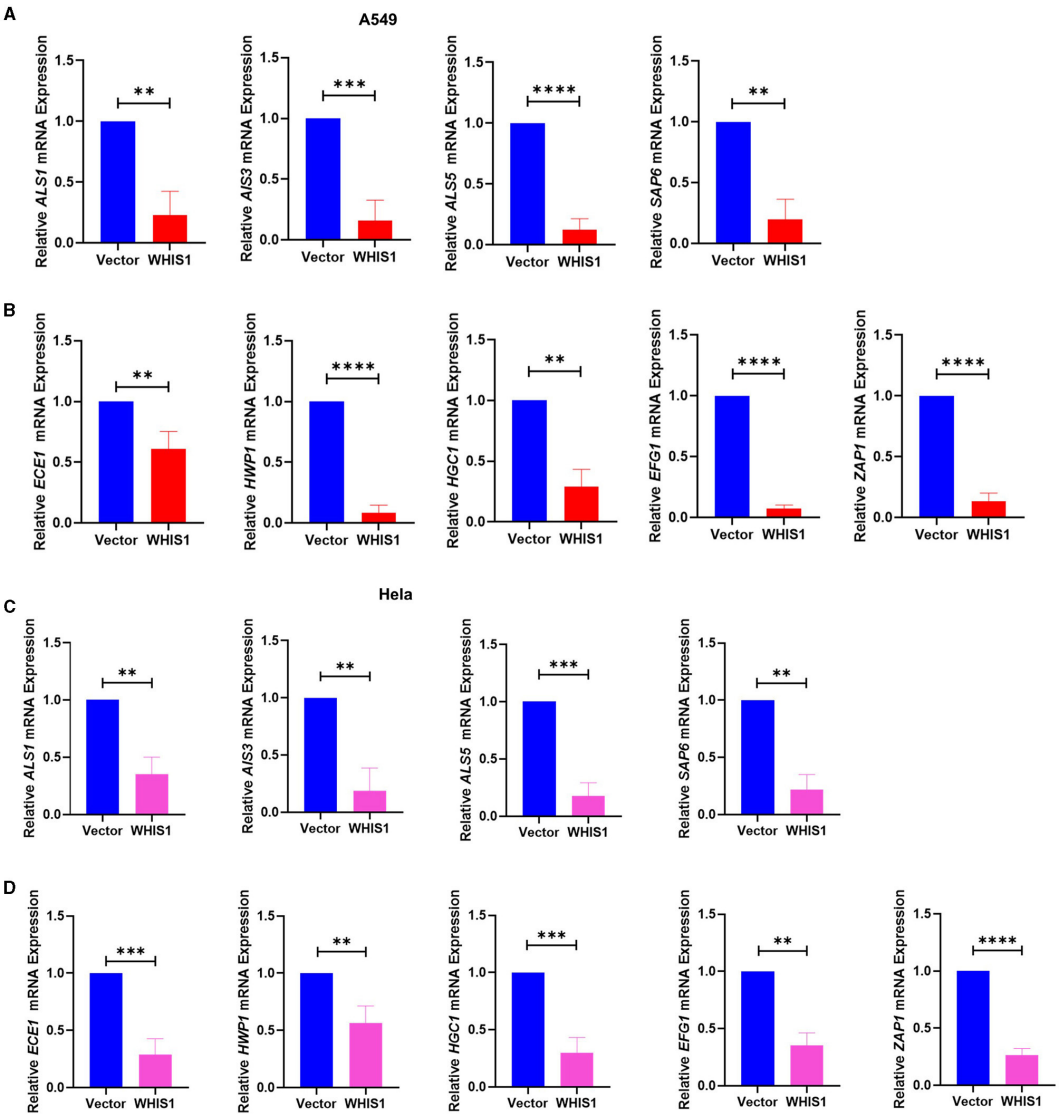
WHIS1 inhibits *Candida albicans* hyphae- and biofilm-related gene expression in A549 **(A**, **B)** and HeLa **(C**, **D)** cells. Twenty-four hours after transfection with WHIS1, A549 or HeLa cells were infected with *C. albicans* at an MOI of 20 for 8 h. Total samples (supernatant + attached cells) were subsequently collected for RNA extraction. The adhesion-related factors *ALS1, ALS3, ALS5*, and *SAP6* **(A**, **C)** and key mycelium formation factors (*ECE1, HWP1, HGC1, EFG1*, and *ZAP1*) **(B**, **D)** were detected by real-time PCR. 18S rRNA was used as the internal reference gene. Statistical analyses were performed with an unpaired *t*-test, and the results are presented as the means ± standard deviations (*n* = 3). **P < 0.01, ***P < 0.001, and ****P < 0.0001 vs. the vector group.

Mycelial cells are a pathogenic form of *C. albicans* and are controlled by several genes (Fan et al., 2013; Kumar et al., 2015; Liu et al., 2021). To further elucidate the inhibitory mechanisms of WHIS1 against *C. albicans*, the key factors involved in the effects of WHIS1 treatment on A549 and HeLa cells were determined (Figure 9). According to the changes in gene expression, the expression of agglutinin-like sequence-encoding genes (*ALS1, ALS3*, and *ALS5*), which promote adhesion, and *SAP6*, a secreted aspartyl proteinase that participates in maintaining the cell surface integrity of *C. albicans*, were strongly downregulated in A549 cells (Figure 9A). Furthermore, several key mycelium formation-related factors (*ECE1, HWP1, HGC1, EFG1*, and *ZAP1*) of *C. albicans* were also significantly downregulated (Figure 9B). To further verify this finding, similar results were obtained with *C. albicans*-infected HeLa cells (Figures 9C, D).

**Figure 10:**
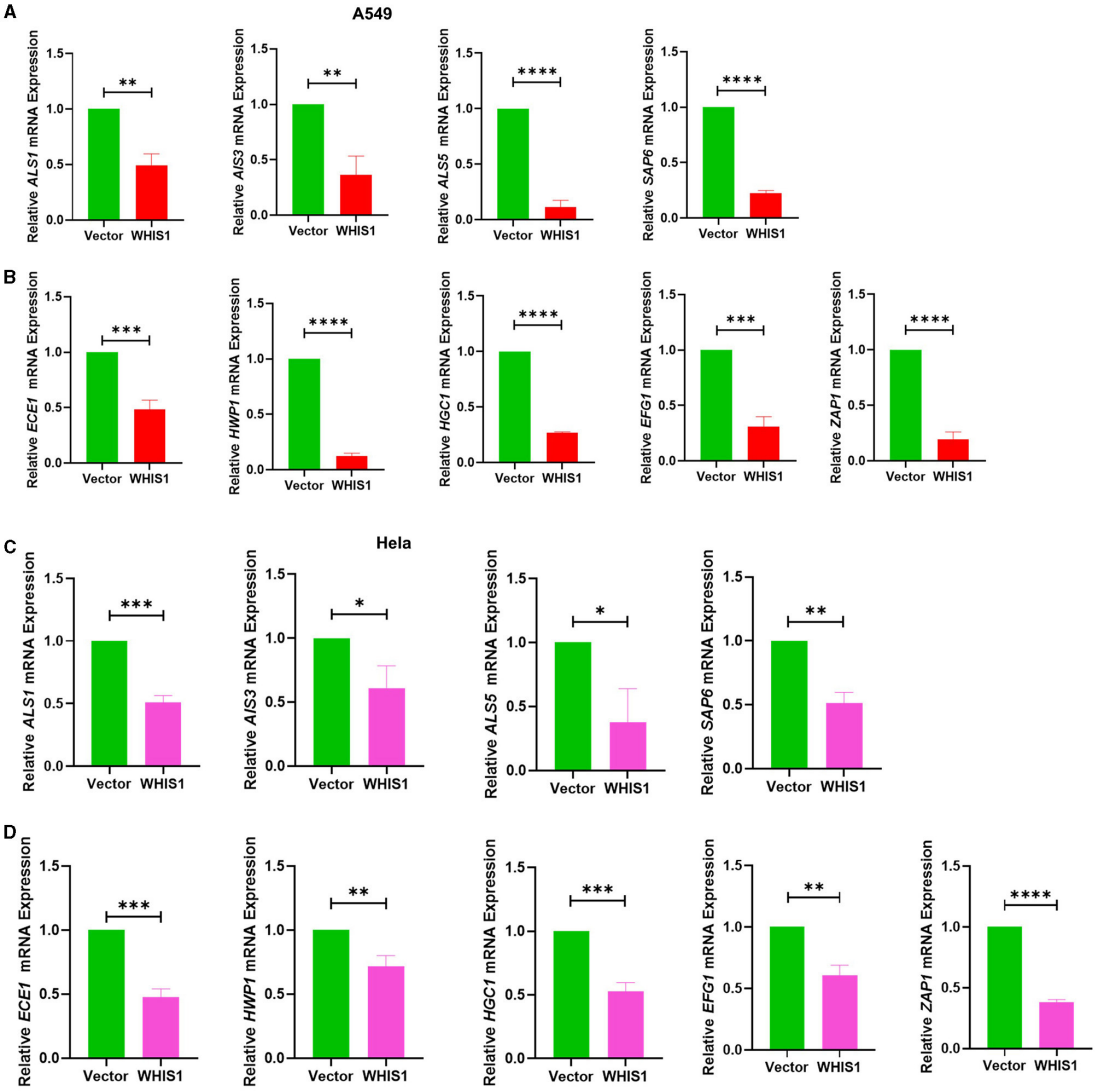
WHIS1 inhibits *Candida albicans* hyphae- and biofilm-related gene expression in A549 **(A**, **B)** and HeLa **(C**, **D)** cells. Twenty-four hours after transfection with WHIS1, HeLa cells were infected with *C. albicans* at an MOI of 20 for 8 h. The supernatant was discarded, and only the attached cells were collected for RNA extraction. The adhesion-related factors *ALS1, ALS3, ALS5*, and *SAP6* **(A**, **C)** and key mycelium formation factors (*ECE1, HWP1, HGC1, EFG1*, and *ZAP1*) **(B**, **D)** were detected by real-time PCR. 18S rRNA was used as the internal reference gene. Statistical analyses were performed with an unpaired *t*-test, and the results are presented as the means ± standard deviations (*n* = 3). *P < 0.05, **P < 0.01, ***P < 0.001, and ****P < 0.0001 vs. the vector group.

To determine the depth to which *C. albicans* invade host cells, the intracellular conditions were analyzed. The same results were observed with intracellular *C. albicans*-infected A549 cells (Figures 10A, B), and the results were verified with *C. albicans*-infected HeLa cells (Figures 10C, D). These data suggest that WHIS1 inhibits *C. albicans* invasion into A549 and HeLa cells by affecting hyphal formation and adhesion-related gene expression both extracellularly and intracellularly.

## Discussion

Vaccination causes substantial pain in women (Hong et al., 2022; Rosati et al., 2022; Agrawal et al., 2023). *C. albicans*, as *one* of the main causative pathogens, can form hyphae and biofilms for long-term resistance and can also cause severe infection (Hameed and Fatima, 2013; Mayer et al., 2013; Naglik et al., 2019). Herein, we identified an insect-SVWC peptide, whole-housefly larvae insect SVWC peptide 1 (WHIS1), via a microbe-stimulated transcriptomic screen of housefly larvae. Here, we cloned WHIS1, modeled its structure, prokaryotically expressed the encoded protein, and tested its antimicrobial activity. Heterologous expressed MdWHIS1 clearly inhibited hyphal formation in *C. albicans*-infected A549 and HeLa cells, and an analysis of its expression pattern showed that WHIS1 was mainly expressed in the fat body and salivary glands of housefly larvae. Moreover, WHIS1 expression was rapidly upregulated by microbial stimulation in houseflies. Furthermore, infection experiments demonstrated that MdWHIS1 reduced the density and hyphae density of *C. albicans* in A549 and HeLa cells. To further elucidate the underlying mechanism involved, the gene expression patterns were assessed, and the results showed that WHIS1 downregulated all hyphal formation-related factors both extracellularly and intracellularly in A549 and HeLa cells. These data showed that WHIS1 inhibits *C. albicans* invasion into epithelial cells by affecting hyphal formation and adhesion factor-related gene expression. This study explored the potential mechanism of the insect SVWC peptide, WHIS1, against *C. albicans* and enhanced the understanding of the antifungal functions of insect SVWC members.

Houseflies live in complex environments due to their powerful innate immune system. AMPs play an important role as antimicrobial agents in the environment (Gao et al., 2015; Wang et al., 2021b, 2022b). WHIS1 expression was first found to be significantly upregulated through a screen of the microbe-stimulated 3rd-instar housefly larva transcriptome (Supplementary Figure 1). A protein BLAST search against the NCBI database showed that the targeted homologs of this protein belonged to insects (Supplementary Figure 2 and Supplementary Excel 1). All the homologs were putative or uncharacterized. Thus, housefly WHIS1 was cloned for the first time and submitted to the NCBI GenBank under project SUB11062418 (Figure 1). However, several members of the SVWC family have been found in arthropods (*M. nipponense* and *E. sinensis*) and function as PRRs (Qin et al., 2020, 2022). Simultaneously, the SVWC peptide *M. japonicus* Vago functions as an IFN analog with anti-WSSV activity via the JAK-STAT signaling pathway (Gao et al., 2021). Moreover, *D. melanogaster* Vago, an SVWC member, reportedly encodes an antiviral peptide (Gao et al., 2021), and *C. quinquefasciatus* Vago functions as an IFN analog with anti-WNV activity (Paradkar et al., 2012). This finding indicates that WHIS1 could function as a PRR or IFN analog. To address this question, sequence alignment was performed, and a phylogenetic tree was constructed. The amino acid sequence of MdWHIS1 is quite different from those of DmVago, CqVago, and arthropod SVWCs (Figure 2A). Furthermore, the genes also clustered in different branches of the phylogenetic tree (Figure 2B), which indicates that these genes show marked sequence differences, apart from the eight conserved cysteines in the SVWC domain (Figures 1–3). Intriguingly, Zhu et al. identified 29 SVWC members in houseflies as AMPs through bioinformatics methods (Qi et al., 2021). Thus, WHIS1 may have different functions although it belongs to the SVWC superfamily.

To further verify the transcriptome of WHIS1, its expression pattern was analyzed. WHIS1 could be upregulated by microbial stimulation and was expressed mainly in the fat body and salivary gland of 3rd-instar housefly larvae. This finding indicates that WHIS1 plays a pivotal role in antimicrobial defense in the host (Figure 4).

After determining the WHIS1 sequence, we used several methods to verify its function. First, we heterologously expressed 100 amino acids without the first 20 amino acids (the signal peptide). To analyze the anti-*C. albicans* activity of WHIS1, the WHIS protein was prepared using a prokaryotic expression method (Figure 5). Obtaining soluble WHIS1 protein posed a challenge due to the consistent formation of inclusion bodies in the initial experiments. Thus, we attempted to optimize the expression conditions, and after refolding the inclusion bodies by dialysis, we obtained some prokaryotically expressed WHIS1 protein. After purification with a His binding kit, an ultrafiltration tube was utilized to filter the elution mixture. MIC and time–growth curve tests were carried out with the purified prokaryotically expressed WHIS1 protein (Supplementary Table 1 and Supplementary Figure 3). The data showed that the prokaryotically expressed proteins inhibited the growth of *C. albicans*. The MIC was 0.011 mmol/L. As shown using ExPASy (https://web.expasy.org/cgi-bin/protparam/protparam), the molecular weight of WHIS1 (containing the signal peptide) was found to be 13,353.79, and the theoretical pI was calculated to be equal to 7.52. In the prokaryotic expression experiment, WHIS1 without its signal peptide was fused with His Taq. Thus, we also detected the protein by Western blotting with anti-His antibody (Figure 5D). The results showed that prokaryotically expressed WHIS1 hybridized with the band at 12.2 kDa. Moreover, WHIS1 comprised the key band. Furthermore, even some protein bands were found in the Coomassie-stained SDS–PAGE gel, and the purified sample protein was filtered by Millipore ultrafiltration devices (tube + membrane chips). Thus, this process prevents other impurity proteins from exerting further influence in subsequent experiments. The sample will be more pure with WIHS1 protein. However, this method used for WHIS1 protein expression is time-consuming and complex, especially due to the large number of inclusion bodies. Thus, a better protein production method is needed for WHIS1.

Subsequently, the eukaryotic expression method was used in this study. Moreover, heterologous expression of WHIS1 prevents the effects of the WHIS1-mediated signaling pathway in insects (if present) (Wang et al., 2017). Therefore, WHIS1 will function by directly affecting other cells. Furthermore, many insect defensins or AMPs are glycosylated. In some cases, glycosylation is necessary for full activity. Prokaryotic expression is unlikely to allow for glycosylation. To avoid this effect, we are interested in testing the eukaryotic expression method. The WHIS1 ORF with a signal peptide coding sequence was cloned and inserted into the eukaryotic overexpression vector PcDNA3.1(+). The construct was then transfected into A549 cells, and after 24 h, the cells were infected with *C. albicans* for 8 h prior to the invasion experiment (Figure 6). Transfection with the empty vector served as a control. The final CFUs of *C. albicans* in the supernatant and intracellular space were calculated. The WHIS1-transfected group showed decreased *C. albicans* counts after infection with different MOIs and in the supernatant or intracellular space of A549 cells (Figures 6A, B). To learn the cytotoxic effects of Amphotericin B to A549 cells, the CCK8 method of cell viability assay was performed. It was shown that 30–45 μg/ml of amphotericin B did not affect the viability of A549 cells (Supplementary Figure 4A). But as time extended, the toxic effect on the cell was constantly increasing (Supplementary Figures 4B–D).

The expression of WHIS1 in A549 cells post-transfection and post-*C. albicans* infection was also detected at different time points. These data showed that the WHIS1 expression level was relatively high at different time points (Figures 6E, F). To further demonstrate the function of WHIS1, all the assays were re-performed using HeLa cells, and the results were similar to those obtained with A549 cells (Figures 6C, D, G, H). PcDNA3.1 plasmid is a commercially available eukaryotic expression vector that has been used in various previous studies. In general, this plasmid can be transfected using Lipofectamine transfection regent into eukaryotic cells such as A549, HeLa, and 293T cells. Its transfection can induce over expression of the target gene. RNA was isolated without DNA and plasmid. We also detected the change in the WHIS1 expression level by using real-time quantitative PCR (Figures 6E–H). It also showed that WHIS1 was highly expressed for a long period. In addition, as the prokaryotically expressed WHIS1 protein directly adds to the A549 cells, it also shows an inhibition effect on *C. albicans* invasion of A549 (Supplementary Figure 5). These data demonstrated that heterologous eukaryotic expression of WHIS1 plays a pivotal role in helping host cells resist *C. albicans* invasion.

Hyphal formation is a key factor in *C. albicans* invasion into host cells (Kadosh, 2016; Jenull et al., 2017; Liu et al., 2021). *C. albicans* acquires infectivity during its transformation from yeast to hyphae stage (Lee et al., 2017; Kim et al., 2019; Liboro et al., 2021). In addition, the hyphae formation will promote the formation of biofilm of *C.albicans* (Kim et al., 2019, 2022; Liboro et al., 2021). Thus, several anti-*C. albicans* agents are targeting the hyphae formation to inhibit its biofilm formation (Lee et al., 2018; Kim et al., 2019, 2022). As shown in Figures 7, 8, WHIS1 reduced the cell density of *C. albicans* and inhibited hyphal formation in A549 and HeLa cells. The long and extensive hyphae of *C. albicans* formed a network in the vector group. However, the growth of *C. albicans* hyphae in A549 cells was inhibited in the WHIS1 group. WHIS1 shortened the length of hyphae and reduced the number of *C. albicans* aggregates. Furthermore, to determine the invasion conditions of *C. albicans* on host cells after WHIS1 treatment, the total supernatant (full view; Figures 7A, D), discarded supernatant (cell surface; Figures 7B, E), and cell digest obtained with ET and Triton X 100 (intracellular; Figures 7C, F) were collected. WHIS1 inhibited the hyphal growth of *C. albicans* invasion into A549 cells (Figures 7C, F). Furthermore, these results were verified in *C. albicans*-infected HeLa cells (Figures 8C, F). These results indicated that WHIS1 can inhibit the density and mycelial growth of *C. albicans*. To further investigate the underlying molecular mechanism, the gene expression changes were subsequently observed under these conditions.

*C. albicans* exhibits many virulence factors that cause disease, including the morphological transition between yeast and hyphal forms, the expression of adhesins and invasins on the cell surface, the formation of biofilms, phenotypic switching, and the secretion of hydrolytic enzymes (Nobile et al., 2009; Buu and Chen, 2013; Hameed and Fatima, 2013; Moyes et al., 2015; Gulati and Nobile, 2016). Some of these pathogeneses are associated with certain gene families, particularly the agglutinin-like sequence (*ALS*) family (Fan et al., 2013; Roudbarmohammadi et al., 2016). The formation of mycelium by *C. albicans* from yeast leads to increased pathogenicity and toxicity to host cells and plays an important role in the invasion ability of *Candida* species (Fan et al., 2013; Mayer et al., 2013; Moyes et al., 2015; Wongsuk et al., 2016; Wagner et al., 2022). In addition, *C. albicans* more easily adheres to substrates and forms biofilms, leading to long-term residence at the host infection site in the host and making its eradication difficult (Mayer et al., 2013; Moyes et al., 2015; Gulati and Nobile, 2016). *ALS1, ALS3*, and *ALS5* promote the adhesion of *C. albicans* (Fan et al., 2013; Roudbarmohammadi et al., 2016; Chevalier et al., 2019). *ALS3*, a cell surface glycoprotein, promotes adhesion and biofilm formation and affects *ALS1* and *ALS5. ALS3* mediates binding to host ligands (such as E-cadherin on epithelial cells and N-cadherin on endothelial cells), thereby triggering engulfment of the fungal cell into the host cell (Cleary et al., 2011; Fan et al., 2013; Roudbarmohammadi et al., 2016).

*SAP6*, a secreted aspartyl proteinase, participates in maintaining the cell surface integrity of *C. albicans*. Following adhesion to host cell surfaces and hyphal growth, *C. albicans* hyphae can secrete hydrolases, such as Sap6, which have been proposed to facilitate active penetration into these cells. In addition, Sap6 is thought to enhance the efficiency of extracellular nutrient acquisition (Buu and Chen, 2013; Kumar et al., 2015; Jenull et al., 2017). Moreover, the levels of extent of cell elongation 1 (*ECE1*) and hyphal wall protein 1 (*HWP1*), which are hypha-specific genes and hyphal morphological switches, depend on hyphal formation, and the expression of both of these genes is regulated by *EFG1* (Nobile et al., 2006; Fan et al., 2013; Richardson et al., 2018; Liu et al., 2021). In addition, *HGC1* and *EFG1*, which are major activators of hyphal development, regulate hyphal-inducing signals. *HGC1* encodes a protein involved in regulating mycelial growth. The expression of this protein is correlated with the growth and extension of hyphae. Hgc1 plays a role in polarized growth and represses cell separation from hyphae (Fan et al., 2013; Panariello et al., 2017; Desai et al., 2018). *ZAP1* governs the balance between hyphal cells and yeast cells in the biofilms of *C. albicans* (Nobile et al., 2009; Ganguly et al., 2011). Together, the results indicate that transition to the hyphal form and adherence cause damage to the host mucosa via the combined action of secreted aspartyl proteases and phospholipases, facilitating the invasion of the epithelium by the organism (Fan et al., 2013; Desai et al., 2018).

To further elucidate the anti-*C. albicans* mechanism of WHIS1, these key hyphal formation factors of *C. albicans* were assessed. All *C. albicans* factors were significantly downregulated in A549 cells after WHIS1 treatment (Figures 9A, B). To further verify the underlying mechanism, HeLa cells, a cervical carcinoma cell line, were selected to mimic *C. albicans* infection. Intriguingly, WHIS1 also inhibited hyphal formation by limiting the expression of hyphal factors in infected cells (Figures 9C, D).

To investigate the role of gene expression of extracellular and intracellular *C. albicans* in host cell invasion, the expression of these factors was measured under different conditions, as described for the hyphal assay. Furthermore, in both the total samples (supernatant + intracellular), which represent all *C. albicans* cells, and the intracellular samples, which represent *C. albicans* cells inside A549 cells, the levels of all the factors were decreased in the WHIS1 group (Figures 10A, B). In addition, both hyphal formation and adhesion factors were downregulated in the intracellular and extracellular *C. albicans*-infected HeLa cell models (Figures 10C, D). Based on the results of this study, it is reasonable to speculate that WHIS1 disrupts the invasion ability of *C. albicans* by inhibiting the formation of hyphae and adhesion factor expression. In contrast to other SVWC members, WHIS1 has antifungal functions as an SVWC peptide in insects.

## Conclusion

In general, our results demonstrated that WHIS1 inhibits *C. albicans* invasion into host cells by affecting hyphal formation and adhesion-related gene expression. These findings increase the understanding of the functions of SVWC members, especially in insects. This study identified a housefly peptide as a novel drug candidate and thus provides a theoretical basis for the clinical treatment of *C. albicans* infections.

## Data availability statement

The original contributions presented in the study are publicly available. This data can be found at: https://www.ncbi.nlm.nih.gov; OQ267401-OQ267401.

## Ethics statement

No animal studies are presented in this manuscript.

## Author contributions

MC: Conceptualization, Data curation, Formal analysis, Investigation, Methodology, Resources, Validation, Visualization, Writing—original draft, Writing—review & editing. W-KH: Conceptualization, Data curation, Formal analysis, Investigation, Methodology, Software, Validation, Writing—review & editing. YY: Conceptualization, Data curation, Formal analysis, Investigation, Methodology, Writing—review & editing. S-MW: Conceptualization, Data curation, Investigation, Methodology, Writing—review & editing. Y-XY: Conceptualization, Methodology, Project administration, Resources, Supervision, Validation, Visualization, Writing—review & editing, Investigation. W-XL: Conceptualization, Data curation, Investigation, Methodology, Writing—review & editing. GL: Conceptualization, Methodology, Resources, Supervision, Writing—review & editing, Investigation. S-FW: Conceptualization, Resources, Supervision, Validation, Writing—review & editing, Investigation. HZ: Methodology, Resources, Supervision, Validation, Writing—review & editing, Investigation. H-ML: Funding acquisition, Methodology, Resources, Supervision, Validation, Writing—review & editing, Investigation. BW: Conceptualization, Funding acquisition, Investigation, Methodology, Project administration, Resources, Software, Supervision, Validation, Visualization, Writing—original draft, Writing—review & editing.

## Supplementary material

Supplementary Figure 1 Fold changes in the expression of WHIS1 in response to different stimuli identified from transcriptomic data.

Supplementary Figure 2 Phylogenetic dendrogram of WHIS1 homologs identified via a BLAST search of the NCBI protein database A phylogenetic tree was constructed using proteins identified from a BLAST search of the NCBI with the WHIS1 amino acid sequence. The top 119 homologous genes were visualized via the NCBI BLAST Tree View **(A)**. WHIS1 is included in the yellow section. WHIS1 and the nine most similar homologs are shown in **(B)** and were visualized using NCBI BLAST Tree View with the neighbor-joining Kimura model (protein).

Supplementary Figure 3 The time–growth curve of *C. albicans* after treatment with prokaryotically expressed WHIS1 or its mutant. The strains were treated with different concentrations of WHIS1 or its mutant for 24 h, and the OD_595_ value was measured with a microplate reader at various time points was measured with a microplate reader. Time–growth curves of *C. albicans* **(A)** with WT WHIS1 or its mutant **(B)** were drawn, *n* = 3.

Supplementary Figure 4 Toxic Assay of Amphotericin B to A549 cells. Cell viability of different concentrations of amphotericin B on A549 cells for 20 min **(A)**, 40 min **(B)**, 1 h **(C)**, and 24 h **(D)**.

Supplementary Figure 5 Effect of prokaryotic WHIS1 inhibits C. albicans invasion of A549 cells. The A549 cells were seeded into 24-well culture plates (8 × 10^4^ cells/well) overnight at 37°C in an atmosphere with 5% CO_2_. Then prokaryotically expressed recombinant WHIS1 was diluted to a final concentration of 0.011 mM (150 μg/ml) by a sterile RPMI 1640 medium. C. albicans suspensions and WHIS1 were added to each well. The A549 cells were infected for 8 h at 37°C and 5% CO_2_. Following incubation, each well was washed twice with 500 μl of PBS, and 1640 containing 40 μg/ml of amphotericin B was added to kill C. albicans outside the monolayer cells. The plate was then incubated at 37°C in 5% CO_2_ for 20 min and washed twice with 500 μl of PBS. The monolayer cells were digested with 100 μl of 0.25% trypsin-0.53 mM EDTA and subsequently lysed with 500 μl of 0.1% Triton X-100 for 10 min in a cell incubator, and the *C. albicans* was subsequently plated on PDA plates to enumerate the intracellular fungi (CFU). Three wells were used for each tested group, *n* = 3.

Supplementary Table 1 Anti-*C. albicans* activity of prokaryotically expressed WHIS1 protein.

Supplementary Table 2 List of primers used for *WHIS1*.

Supplementary Table 3 List of primers used for the real-time PCR assay of *C. albicans* biofilm-related genes.

Supplementary Excel 1 Alignment of proteins identified through a BLAST search of NCBI based on WHIS1.
