# Supplementary material for: Heterologous expression of the insect SVWC peptide WHIS1 inhibits Candida albicans invasion into A549 and HeLa epithelial cells
Source: Front Microbiol. 2024 May 30;15:1358752. doi: 10.3389/fmicb.2024.1358752 (PMC11169590; doi:10.3389/fmicb.2024.1358752)
Supplement: Supplementary Figure 1 — Fold changes in the expression of WHIS1 in response to different stimuli identified from transcriptomic data. [file Data_Sheet_1.docx]

**Supplementary Table 1 Antimicrobial effect of WHIS Protein (MIC _mmol/L_)**

|  | (MIC mmol/L) | | (MFC mmol/L) | |
| --- | --- | --- | --- | --- |
|  | Wide Type | Mutant | Wide Type | Mutant |
| ***C. albicans*** | 0.011 | 0.011 | 0.022 | **----** |
| ***S. aureus*** | 0.011 | 0.011 | **----** | **----** |
| ***E. coli*** | 0.022 | **----** | **----** | **----** |

**Supplementary Table 2** Primer list of *WHIS1* used here.

| Gene | Sequence (5' to 3') | Specific bp | Usage |
| --- | --- | --- | --- |
| WT- *WHIS1* F | CGGGGTACCGCATTCAATACGTTTGTGATATTTAACG | 26 | Clone/ eukaryotic expression |
| WT-*WHIS1* R | TTTATAGCGGCCGCGTAGGTGGAGTCCATGCAATTTTATTCC | 20 | Clone/ eukaryotic expression |
| P-WT- *WHIS1* F | CGCCATATGTACAGCTTTTTAATACCGGCA | 24 | Prokaryotic expression |
| P-WT-*WHIS1* R | CCGCTCGAGGGCATACACTCCCGTTTCCAG | 25 | Prokaryotic expression |
| P-Mutant-*WHIS1* F | CGCCATATGTACAGCTTTTTAATACCGACAAGAC | 26 | Prokaryotic expression |
| P-Mutant-*WHIS1* R | CCGCTCGAGGGCATACACTCCCGTCTCCAG | 28 | Prokaryotic expression |
| R-WT- *WHIS1* F | GCACCAATCGCATTCCAAG | 19 | Real time - PCR |
| R-WT-*WHIS1* R | CAGATGATGCACAGTCAAATCCTAAG | 26 | Real time - PCR |

**Supplementary Table 3** Realtime PCR primer list of *C.albicans* biofilm related genes.

| Gene | Sequence (5' to 3') | bp |
| --- | --- | --- |
| *ALS1* F | TTCTCATGAATCAGCATCCACAA | 23 |
| *ALS1* R | CAGAATTTTCACCCATACTTGGTTTC | 26 |
| *ALS3* F | GTTACATTTAATGATGGTGGCAAG | 24 |
| *ALS3* R | CATAAGTGTTAGCGAATCCCATTG | 24 |
| *ALS5* F | CTGCCGGTTATCGTCCATTTA | 21 |
| *ALS5* R | TTGATACTGGTTATTATCTGAGGGAGAAA | 29 |
| *ECE1* F | CCAGAAATTGTTGCTCGTGTTG | 22 |
| *ECE1* R | CAGGACGCCATCAAAAACG | 19 |
| *HWP1* F | CCCACAGGTAGACGGTCAAGG | 21 |
| *HWP1* R | GGTTGAGGTGGATTGTCGC | 19 |
| *EFG1* F | CAGTATGGTCAGTATAATGCTGGTAAGAA | 29 |
| *EFG1* R | TTGTTGTTGCTGTTGGTATGGATATGATGATG | 32 |
| *SAP6* F | TTAGATTCGGCAGTTGGATCATC | 23 |
| *SAP6* R | TCTCCAGGGTTTGTTACCTTAGACTT | 26 |
| *HGC1* F | AATTGAGGACCTTTTGAATGGAAA | 24 |
| *HGC1* R | AAAGCTGTGATTAAATCGGTTTTGA | 25 |
| *ZAP1* F | ATCTGTCCAGTGTTGTTTGTA | 21 |
| *ZAP1* R | AGGTCTCTTTGAAAGTTGTG | 20 |
| *18S* F | AAACGGCTACCACATCCAAG | 20 |
| *18S* R | CCAAGCCCAAGGTTCAACTA | 20 |


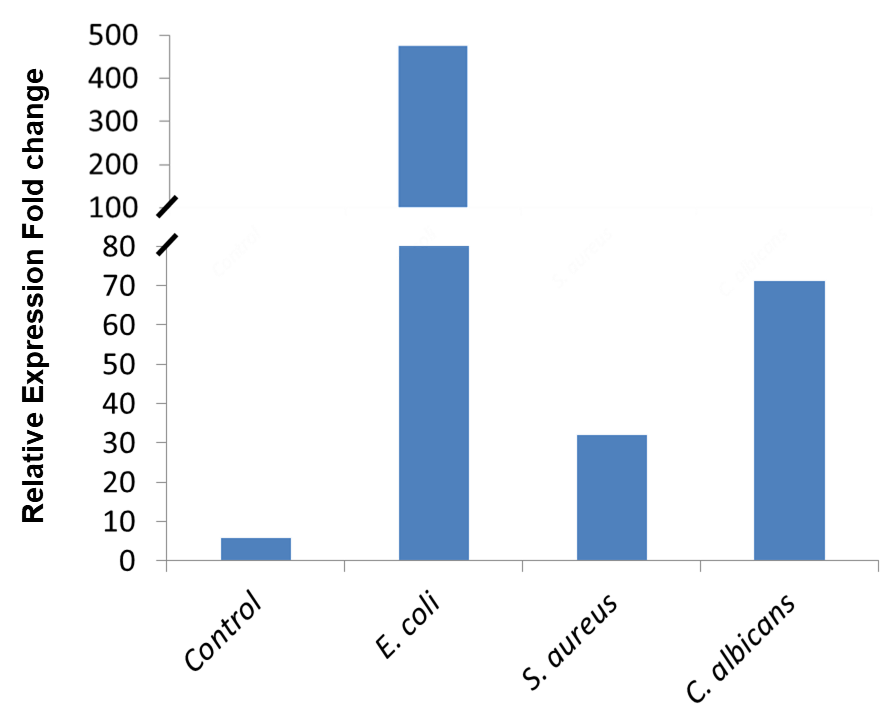


**Supplementary Figure. 1** The fold change in the expression of WHIS1 in response to different stimuli in the transcriptomic data.


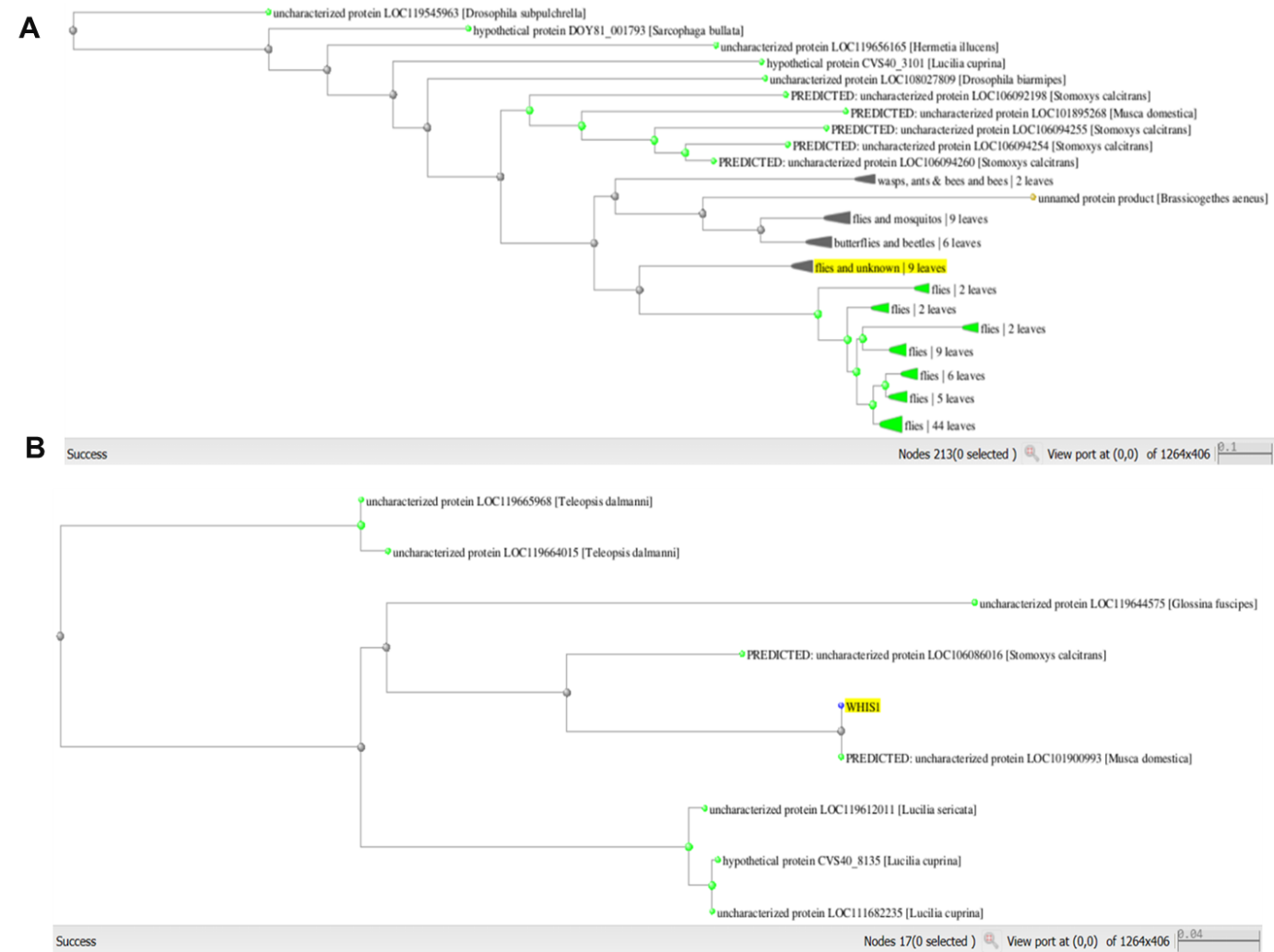


**Supplementary FIGURE 2** Phylogenetic dendrogram of WHIS1 homologues in NCBI Protein BLAST

A phylogenetic tree was constructed in NCBI via Protein BLAST with WHIS1 amino acids. The top 119 homologous genes were visualized in NCBI BLAST Tree View (A). WHIS1 is included in the yellow part. WHIS1 and the 9 most similar homologues are shown in (B), visualized in NCBI BLAST Tree View by a neighbour-joining, Kimura model (protein).


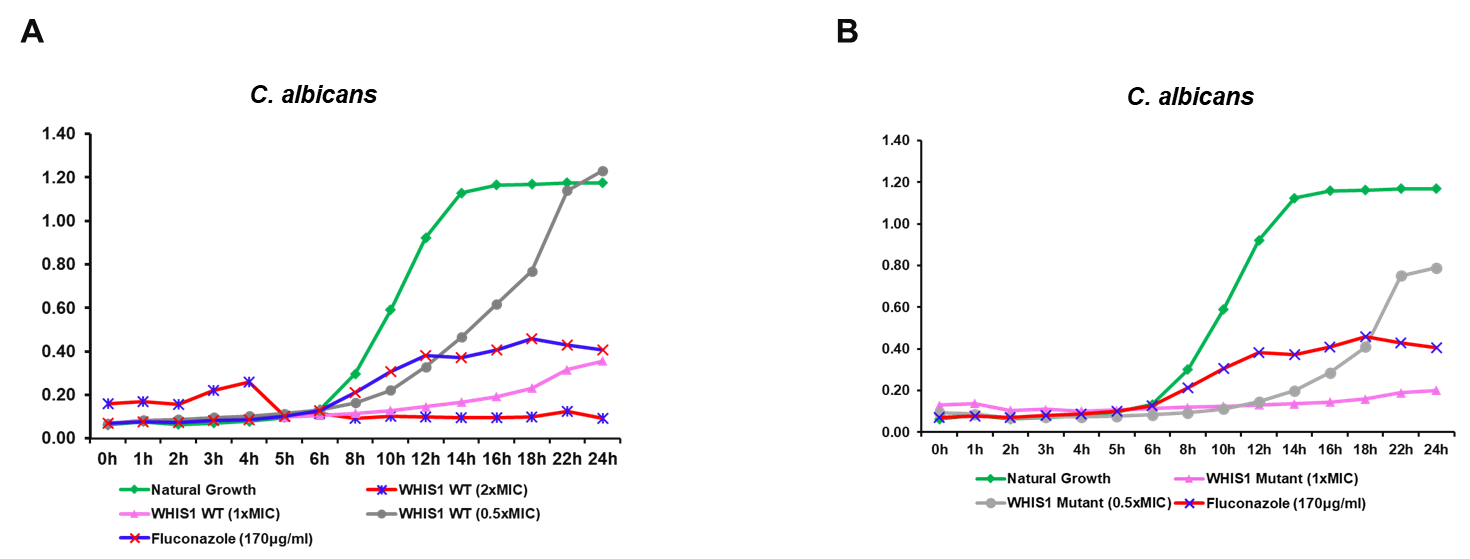


**Supplementary FIGURE 3** Time-growth curve of *C. albicans* upon treatment with prokaryotically expressed WHIS1 or its mutant.

The strains were treated with different concentrations of WHIS1 or its mutant for 24 hours, and the OD_595_ was measured with a microplate reader at each time point. Time-growth curves of *C. albicans* **(A)***,* with WHIS1 WT or its mutant **(B)** were drawn.

**
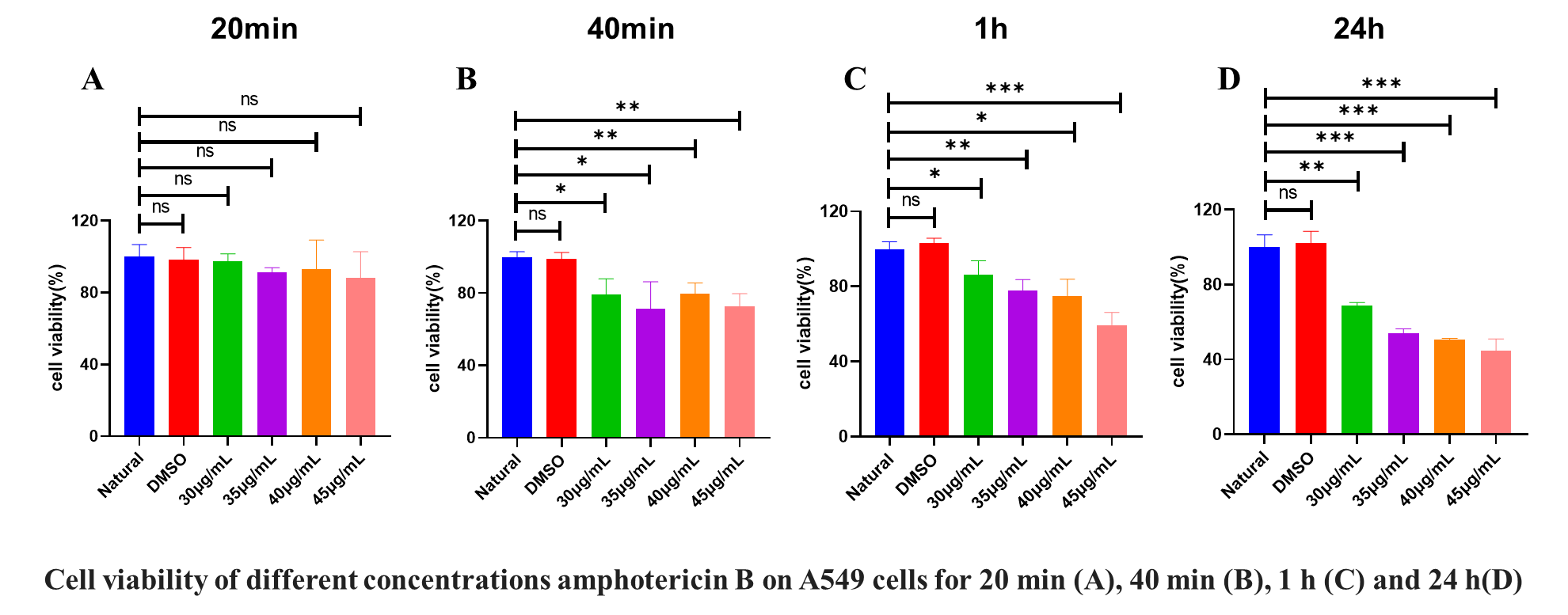
**

**Supplementary FIGURE 4** Toxic Assay of Amphotericin B to A549 cells.

Cell viability of different concentrations amphotericin B on A549 cells for 20 min (A), 40 min (B), 1 h (C) and 24 h(D).

**Supplementary FIGURE 5** Effect of prokaryotic WHIS1 inhibits C. albicans invasion of A549 cells.

The A549 cells were seeded into 24-well culture plates (8×104 cells/well) overnight at 37°C in an atmosphere with 5% CO2. Then prokaryotically expressed recombinant WHIS1 was diluted to a final concentration of 0.011mM (150 μg/ml) by sterile RPMI 1640 medium. C. albicans suspensions and WHIS1 was added to each well. The A549 cells were infected for 8 h at 37°C and 5% CO2. Following incubation, each well was washed twice with 500 μL of PBS, and 1640 containing 40 μg/mL amphotericin B was added to kill C. albicans outside the monolayer cells. The plate was then incubated at 37 °C in 5% CO2 for 20 min and washed twice with 500 μL of PBS. The monolayer cells were digested with 100 μL of 0.25% trypsin-0.53mM EDTA and subsequently lysed with 500 μL of 0.1% Triton X-100 for 10 min in a cell incubator, and the C. albicans was subsequently plated on PDA plates to enumerate the intracellular fungi (CFU). Three wells were used for each tested group. n=3.
